# Transcriptional factor ZMYM3 promotes hepatocellular carcinoma metastasis by upregulating CTTN and inducing invadopodia formation

**DOI:** 10.1038/s41419-026-08506-6

**Published:** 2026-03-03

**Authors:** Fuling Zeng, Zihua Zhang, Tingting Hu, Xin Xia, Da-Lin Lu, Chen Qu, Lu He

**Affiliations:** 1 Department of Pathophysiology, School of Medicine, Jinan University, Guangzhou, Guangdong 510630 China; 2 Department of Laboratory Medicine, Shenzhen Guangming District People’s Hospital, Shenzhen, Guangdong 518000 China; 3 Department of Epidemiology, School of Medicine, Jinan University, Guangzhou, Guangdong 510630 China; 4 Department of Radiotherapy, Affiliated Cancer Hospital & Institute of Guangzhou Medical University, Guangzhou, Guangdong 510095 China

**Keywords:** Liver cancer, Invadopodia

## Abstract

Hepatocellular carcinoma (HCC) is characterized by high invasiveness and metastatic potential, leading to poor prognosis. Therefore, understanding the molecular mechanisms underlying HCC invasion and metastasis is essential for developing effective therapeutic strategies. This study investigates the role of ZMYM3 in HCC invasion and metastasis. Analysis of The Cancer Genome Atlas (TCGA) and Gene Expression Omnibus (GEO) datasets, along with immunohistochemistry, revealed that ZMYM3 is upregulated in HCC tissues and associated with recurrence and poor prognosis. Single-cell sequencing data indicated higher ZMYM3 expression in portal vein tumor thrombus compared to primary lesions, suggesting its involvement in metastasis. Functional assays demonstrated that ZMYM3 enhances HCC cell proliferation, invasion, and metastasis. RNA sequencing identified that ZMYM3 promotes invadopodia formation and epithelial-mesenchymal transition (EMT) in HCC cells. Further chromatin immunoprecipitation sequencing and mechanistic studies showed that ZMYM3 directly binds to the promoter of CTTN, a key gene regulating invadopodia formation, thereby increasing its expression. This upregulation contributes to the enhanced invasive and metastatic capabilities of HCC cells. Our findings identify ZMYM3 overexpression as a predictor of high recurrence risk and poor prognosis in HCC patients. Mechanistically, ZMYM3 promotes invadopodia formation primarily through the upregulation of CTTN, thereby augmenting the invasive and metastatic potential of HCC cells. These results highlight the critical role of ZMYM3 in HCC progression and metastasis.

**Figure:**
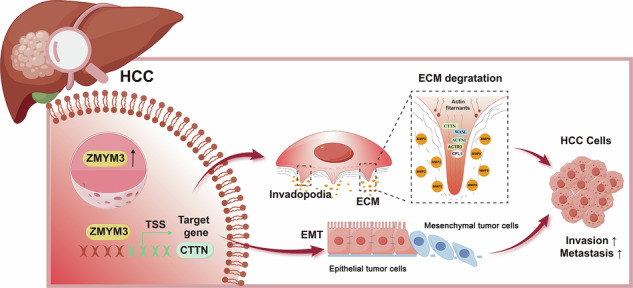
ZMYM3 promotes hepatocellular carcinoma metastasis by upregulating CTTN and inducing invadopodia formation.

ZMYM3 promotes hepatocellular carcinoma metastasis by upregulating CTTN and inducing invadopodia formation.

## Introduction

Primary liver cancer is the sixth most common malignancy and the third leading cause of cancer-related deaths worldwide [1]. Hepatocellular carcinoma (HCC), the predominant form of liver cancer, accounts for over 75% of cases [2]. Due to its insidious onset and highly aggressive, invasive, and metastatic nature, early detection of HCC is often challenging, with many patients diagnosed at advanced stages, thus missing the window for surgical intervention [3]. Even after undergoing surgery or liver transplantation, metastasis and recurrence remain frequent [4]. Therefore, a deeper understanding of the mechanisms driving HCC invasion and metastasis is critical for developing new therapeutic strategies and targets, which could guide clinical management and improve survival outcomes for HCC patients.

Numerous studies have demonstrated that invadopodia play a critical role in the invasion, migration, and dissemination of HCC cells [5]. Invadopodia are dynamic, actin-rich protrusions formed at the leading edge of cells, particularly in tumor cells or other migrating and invasive cells [6]. Their formation involves cytoskeletal reorganization and the mobilization of specific proteins. Once established, invadopodia increase the surface area of tumor cells, facilitating interactions with the extracellular matrix and driving outward protrusion [7]. Additionally, they secrete matrix metalloproteinases (MMP2/MMP9) and interact with cell surface receptors, leading to degradation and remodeling of the extracellular matrix. This process promotes tumor cell penetration through the basement membrane and subsequent invasion into surrounding tissues [8, 9]. Moreover, the formation of invadopodia is closely related to the epithelial-mesenchymal transition (EMT) of tumor cells. EMT refers to the process in which tumor cells lose their epithelial characteristics and acquire mesenchymal traits, during which their ability to form invadopodia is enhanced [10]. The formation of invadopodia is one of the mechanisms through which EMT promotes tumor cells invasion and metastasis.

ZMYM is a member of the zinc finger MYM-type DNA-binding protein family [11], known for its role in regulating gene transcription and chromatin structure [12]. Previous studies have identified ZMYM as an oncogenic driver in several cancers [13–15], where it controls the expression of downstream target genes through transcriptional regulation and is involved in critical biological processes such as cell cycle regulation, DNA damage repair, and histone modification, ultimately promoting tumor progression [16, 17]. However, the specific role of ZMYM3 in hepatocellular carcinoma (HCC) remains unclear.

Through RNA sequencing and chromatin immunoprecipitation sequencing (ChIP-seq), our study revealed that ZMYM3 may directly upregulate CTTN expression, thereby promoting the formation of invadopodia in HCC cells. CTTN is a core regulator of invadopodia formation. It is an important cytoskeletal protein localized in the cytoplasm and at cell–matrix contact sites. Current studies suggest that CTTN is a key regulator of invadopodia formation in tumor cells. During the initiation stage of invadopodia formation, CTTN accumulates at the regions where the plasma membrane contacts the extracellular matrix, recruiting and polymerizing F-actin to form the initiation core of invadopodia. It subsequently mobilizes and organizes other essential proteins to establish mature invadopodia structures. Knockdown of CTTN expression has been shown to suppress invadopodia formation in tumor cells [18]. CTTN is highly expressed in multiple malignancies, including hepatocellular carcinoma (HCC), breast cancer, and oral squamous cell carcinoma, and its expression level is closely associated with tumor invasiveness and metastatic potential [18–20]. Moreover, previous studies have reported that CTTN interacts with the Hepatitis B virus–encoded X protein (HBx) to regulate CREB1 and its downstream target genes, thereby promoting the proliferation and metastasis of HCC cells [21]. However, the mechanisms underlying the high expression of CTTN in tumor cells remain incompletely understood.

In this study, we found that ZMYM3 is significantly overexpressed in HCC and is associated with poor patient prognosis. Notably, we demonstrated that ZMYM3 promotes the invasion and metastasis of HCC cells by upregulating CTTN expression and enhancing invadopodia formation. These findings provide new insights into the molecular mechanisms underlying HCC cell invasion and metastasis.

## Materials and methods

### Clinical samples

The 83 HCC samples and 20 adjacent non-tumor liver tissue samples used in this study were obtained from HCC patients who underwent radical resection at the Affiliated Cancer Hospital of Guangzhou Medical University. Clinical and pathological data were sourced from the hospital’s archives. Ethical approval for the use of these clinical samples was granted by the Ethics Committee of the Affiliated Cancer Hospital of Guangzhou Medical University. All tissue samples were obtained after patients provided informed consent.

### Cell culture

The human HCC cell lines MHCC97H and MHCC97L were sourced from the Shanghai Cell Bank of Chinese Academy of Science (Shanghai, China). The HCCLM3, Hep3B, HepG2, and Huh7 cell lines were obtained from Haixing Biological Company (Guangzhou, China). All cells were confirmed to be mycoplasma-free and verified by short tandem repeat (STR) analysis. Cells were cultured in DMEM (ThermoFisher, Waltham, MA, USA) supplemented with 10% fetal bovine serum and 1% penicillin-streptomycin (ThermoFisher).

### Western blotting

Protein samples were loaded onto an SDS-PAGE gel system for electrophoresis, followed by protein transfer to a PVDF membrane. Membranes were cut according to molecular weight markers and blocked with 5% skim milk for 1 h at room temperature. Primary antibodies were applied and incubated overnight at 4°C. After washing, secondary antibodies were added, followed by ECL reagent (Merck, Darmstadt, Germany) for visualization. Details of the source of used primary antibodies are listed in Supplementary Table 1.

### Immunohistochemistry staining

Formalin-fixed, paraffin-embedded tissue specimens were cut into 4 μm-thick sections. Sections were baked, deparaffinized, and rehydrated according to standard procedures. Antigen retrieval was performed using a sodium citrate buffer, followed by blocking. Primary antibodies were incubated at 4°C overnight, followed by rewarming, washing, and incubation with secondary antibodies. A non-biotin horseradish peroxidase detection system (ZSGB-Bio, Beijing, China) was used to detect the expression level of the protein of interest. Both the extent and intensity of immunostaining were taken into consideration when analyzing the data with intensity scored from 0 (negative) to 3 (strong positive), and extent scored from 0 (10%) to 4 (76–100%). The total score was the sum of both parameters, categorizing samples into high- and low-expression groups. Details of the source of used primary antibodies are listed in Supplementary Table 1.

### Immunofluorescence (IHC) staining

Cells were fixed with 4% paraformaldehyde and permeabilized using 0.5% Triton-X100. After blocking with 5% BSA, the primary antibody was incubated overnight at 4°C. The secondary antibody was applied at room temperature for 1 h, followed by DAPI for nuclear staining. Anti-fluorescence quenching agent was applied to the slides, which were then imaged using confocal and fluorescence microscopy (Leica, Wetzlar, Germany). Details of the source of used primary antibodies are listed in Supplementary Table 1.

### Real-time quantitative PCR (RT-qPCR)

Total RNA was extracted from cells using TRIzol reagent, and RNA concentration was measured with a NanoDrop spectrophotometer (ThermoFisher Scientific). cDNA was synthesized using the PrimeScript RT Master Mix kit (ThermoFisher Scientific), followed by RT-qPCR using the PowerUp SYBR Green Master Mix kit (ThermoFisher Scientific). The relative expression of target genes was calculated using the 2-ΔΔCt method. PCR primers are provided in Supplementary Table 2.

### Cell proliferation assay

A suspension of 3000 cells in 100 μl was seeded into 96-well plates and incubated for 36 h. Then, 100 μl of medium containing 10% CCK-8 reagent ((Dojindo, Kumamoto, Japan)) was added to each well. After 2 h, absorbance was measured at 450 nm. Readings were taken at 0, 24, 48, 72, and 96 h using a microplate reader (ThermoFisher Scientific).

### Colony formation assay

Cells in the logarithmic growth phase were seeded at 500 cells per well in 6-well plates (Nest, China) and cultured for 14 days. Afterward, cells were fixed with methanol and stained with crystal violet. Colonies with more than 50 cells were observed and counted.

### Apoptosis analysis

Cells were digested, collected, and washed. After resuspending in 500 μl of Binding Buffer, 5 μl each of Annexin V-APC and PI (Multi Sciences, Hangzhou, China) was added. After incubating in the dark for 5–10 min, samples were analyzed using a flow cytometer (Beckman Coulter, Brea, CA, USA).

### Cell cycle analysis

Cells were harvested, washed, and fixed. Then, 1 ml of DNA staining solution and 10 μl of permeabilization solution (Multi Sciences) were added, followed by incubation in the dark for 30 min. Flow cytometry was used for analysis.

### Wound healing assay

At 80% confluence, scratches were made in a monolayer of cells in a 6-well plate. After washing with PBS, fresh medium was added, and images were captured at 0, 24, and 48 h to monitor wound closure.

### Transwell migration and Matrigel invasion assays

Cell motility was assessed by cell migration and invasion assays using Transwell chambers (Corning, Corning, NY, USA) with or without Matrigel (BD Biosciences, Franklin Lakes, NJ, USA). For migration, 1 × 10^4^ cells suspended in 100 μl of serum-free medium were seeded into the upper chamber, and 500 μl medium with 20% serum was added to the lower chamber. After 16 or 36 h, cells that migrated were fixed, stained with DAPI, and imaged. For invasion, 8% Matrigel was applied to the upper chamber before seeding cells.

### Nude Mouse Model

Twenty-eight five-week-old male BALB/c nude mice, weighing 18–20 grams, were randomly assigned to different experimental groups for subcutaneous xenograft and lung metastasis models. For subcutaneous xenograft 2×10^6^ logarithmically growing cells, were suspended in 200 microliters of DMEM medium. Subsequently, this suspension was injected into the right flanks of the nude mice. Tumor volumes were measured and documented every three days, and the tumors were excised from the mice, weighed, and photographed four weeks after injection. For the lung metastasis model, 6×10^6^ cells resuspended in 50 microliters of PBS were injected into the tail veins of the nude mice. Six weeks later, all mice were euthanized, and their lungs were resected for the purpose of counting metastatic nodules. The data analysis was blinded to group allocation. The nude mice were sourced from the from Zhejiang Vital River Laboratory Animal Technology Co. Ltd (Zhejiang, China). All animal studies were approved by the Institutional Animal Ethical Committee of the Experimental Animal Center of Jinan University.

### RNA transfection and lentiviral infection

ZMYM3-specific siRNA was transfected into HCC cells using Lipofectamine 3000 reagent (ThermoFisher Scientific) according to the protocol provided by the manufacturer. ZMYM3 overexpression lentivirus (OBIO TECHNOLOGY, Shanghai, China) was used for stable transfection of HCCLM3 cells. siRNA sequences are listed in Supplementary Table 2.

### RNA-seq analysis

Following transfection with siRNA targeting ZMYM3 and negative control siRNA, the HCC cells were subjected to lysis using TRIzol reagent (ThermoFisher Scientific). Total RNA samples were submitted to LC Sciences Biotech (Hangzhou, China) for sequencing. The RNA concentration was quantified using an enzyme-labeled analyzer, and RNA quality was assessed using the Bioanalyzer 2100 system. Paired-end RNA sequencing with a read length of 150 bp (PE150) was performed on the Illumina Novaseq 6000 platform to sequence the purified RNA samples. Differentially expressed genes were identified, and enrichment analyses were conducted using R packages such as “clusterProfiler” and “ggplot2”.

### Chromatin immunoprecipitation

Chromatin Immunoprecipitation (ChIP) experiments were performed following the Merck EZ-Magna ChIP kit protocol (Merck, 17-10086). FLAG-ZMYM3 overexpressing cells were cross-linked with formaldehyde, lysed, and sonicated to obtain chromatin fragments. Immunoprecipitation was conducted with anti-FLAG antibodies (Cell Signaling Technology, Danvers, MA; #14793). Normal IgG (negative control) antibodies were included in the Merck ChIP kit. The precipitated DNA was purified and analyzed by sequencing and qPCR with DNA purified and analyzed. Primer sequences are provided in Supplementary Table 2.

### Dual-luciferase reporter assay

Luciferase reporter vectors containing the wild-type or mutant sequences towards the ZMYM3 binding of the CTTN promoter region were constructed by IGE Bio (Guangzhou, China). The pGL3-basic vector was included as the control. For luciferase activity assays, the control lentivirus-vector control and lentivirus-ZMYM3 overexpressing HCCLM3 cells were seeded in 24-well plates and cultured 24 h before transfection. Then the control or CTTN promoter plasmids were transfected into the indicated cells. After 48 h, the cells were lysed and performed a dual-luciferase reporter assay following the instruction of the Dual Luciferase Reporter Gene Assay Kit (Abbkine Scientific, Wuhan, China). The Firefly and Renilla luciferase activities were detected by a multifunctional microplate reader.

### Cell adhesion assay

Wells of a 96-well plate were coated with fibronectin or collagen I, followed by seeding of 5 × 10^4^ cells. Non-adherent cells were washed away, and adherent cells were quantified using the CCK-8 (Dojindo).

### Cytoskeletal staining and invadopodia detection

Cells were fixed, permeabilized, and blocked, followed by staining with Cortactin and phalloidin. Cells were imaged using a confocal microscope to detect invadopodia.

### Public datasets

Data from The Cancer Genome Atlas (TCGA) and Gene Expression Omnibus (GEO) databases were analyzed using R software. Additional datasets were obtained from the Cistrome Data Browser for further analysis.

### Statistical analysis

Independent t-tests were used to assess significance between two groups, and categorical variables were analyzed using the chi-square test. ANOVA was employed for comparisons across multiple groups. The Kaplan–Meier method was used to determine survival probability, and the log-rank test assessed the differences. All experiments were performed at least three times independently. Data were presented as mean ± SD, and significance was indicated as follows: *P < 0.05, **P < 0.01, ***P < 0.001. All statistical analyses were performed using GraphPad Prism 8.0.

## Results

### The abnormal upregulation of ZMYM3 implies poor prognosis and a heightened invasive capacity in HCC

**Fig. 1:**
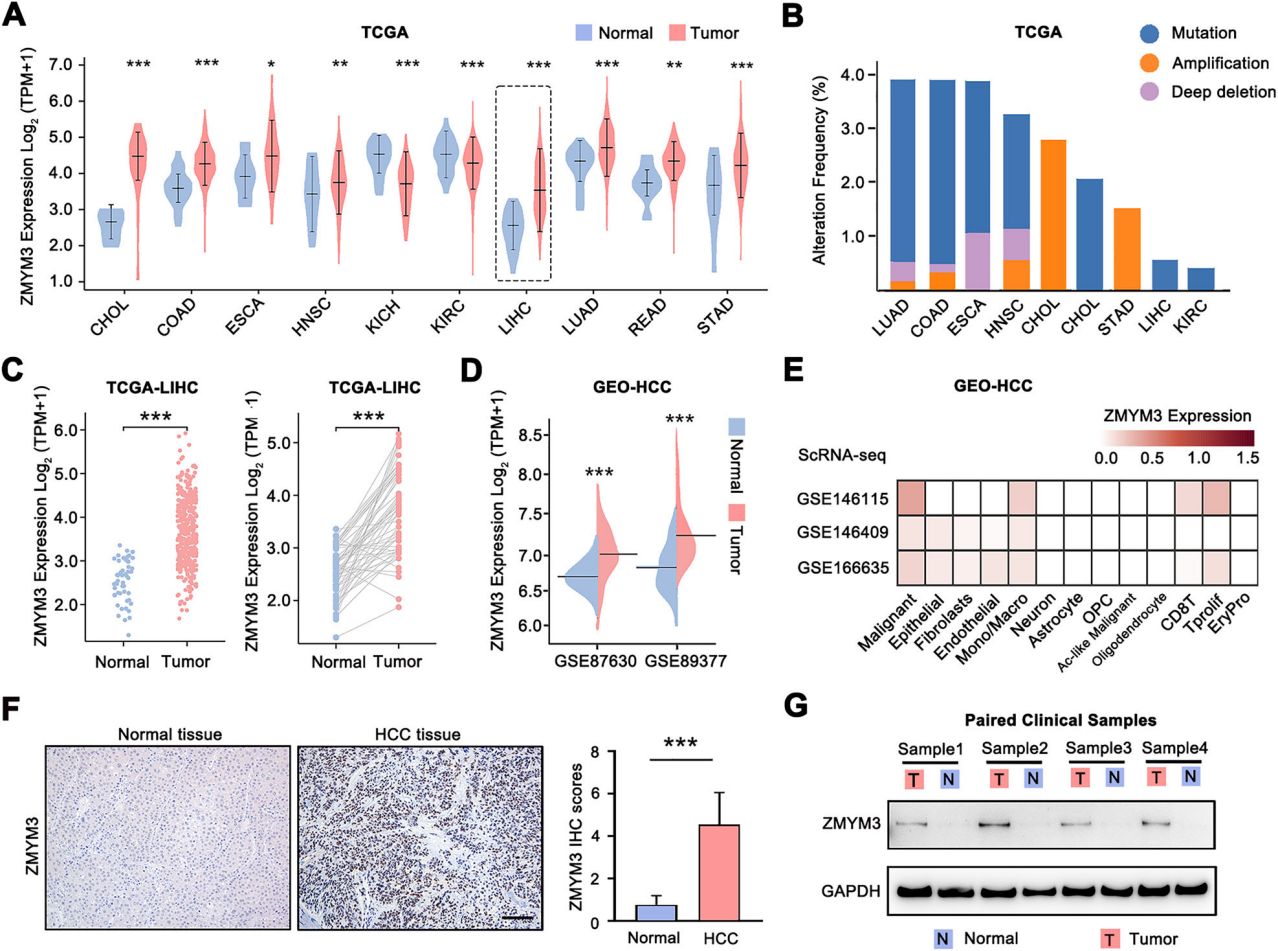
The abnormal upregulation of ZMYM3 in HCC tissues. **A** Differential expression of *ZMYM3* mRNA between various tumors and adjacent normal tissues from TCGA database. **B** The frequency of *ZMYM3* gene alterations in various tumors from the TCGA database. **C** The left panel showed the differential expression of *ZMYM3* mRNA in liver hepatocellular carcinoma (LIHC; 50 normal samples versus 374 tumor samples). The right panel showed the differential expression of *ZMYM3* mRNA in paired cancerous and para-cancerous non-tumor tissues from HCC patients. The data were from TCGA. **D** Differential *ZMYM3* mRNA expression between HCC tissues and adjacent non-tumor tissues from the GEO database. (GSE87630: Normal=30, Tumor= 64; GSE89377: Normal=13, Tumor=94). **E** The expression levels of ZMYM3 in different cells were analyzed by ScRNA-seq data of HCC. **F** Representative IHC staining image and IHC scores of ZMYM3 in HCC tissues (*n* = 83) and normal tissues (*n* = 20). Scale bar, 100 μm. **G** Protein expression of ZMYM3 in paired HCC and non-tumor tissues were detected by Western blot. *P < 0.05; **P < 0.01; ***P < 0.001.

We conducted a pan-cancer analysis using TCGA data and found that ZMYM3 was significantly overexpressed in various tumor tissues compared to adjacent non-tumorous tissues **(**Fig. 1A**)**. Notably, ZMYM3 expression was markedly higher in HCC tissues than in adjacent non-tumorous liver tissues **(**Fig. 1A, **LIHC)**. Genetic alteration analysis revealed a mutation rate of less than 1% for *ZMYM3* in HCC, suggesting that the abnormal upregulation of ZMYM3 was primarily driven by non-mutational mechanisms rather than genetic mutations **(**Fig. 1B**)**. Further validation using data of paired HCC and adjacent non-tumorous tissues from TCGA and GEO datasets (GSE87630 & GSE89377) confirmed the elevated expression of ZMYM3 in HCC tissues **(**Fig. 1C, D). Additionally, single-cell RNA sequencing (ScRNA-seq) data from GEO demonstrated predominant expression of ZMYM3 in HCC cells, as well as in epithelial cells, fibroblasts, endothelial cells, and macrophages, with the highest levels observed in HCC cells **(**Fig. 1E**)**. We also assessed the protein expression of ZMYM3 in HCC tissues. Immunohistochemical (IHC) staining results revealed that ZMYM3 levels in 83 HCC tissues were significantly higher than in 20 normal liver tissues **(**Fig. 1F**)**. Furthermore, Western blot analysis of four paired samples corroborated the upregulation of ZMYM3 protein in HCC tissues compared to adjacent normal tissues **(**Fig. 1G**)**. These findings indicate that ZMYM3 is abnormally upregulated in HCC tissues and suggest its potential role in HCC development.

**Fig. 2:**
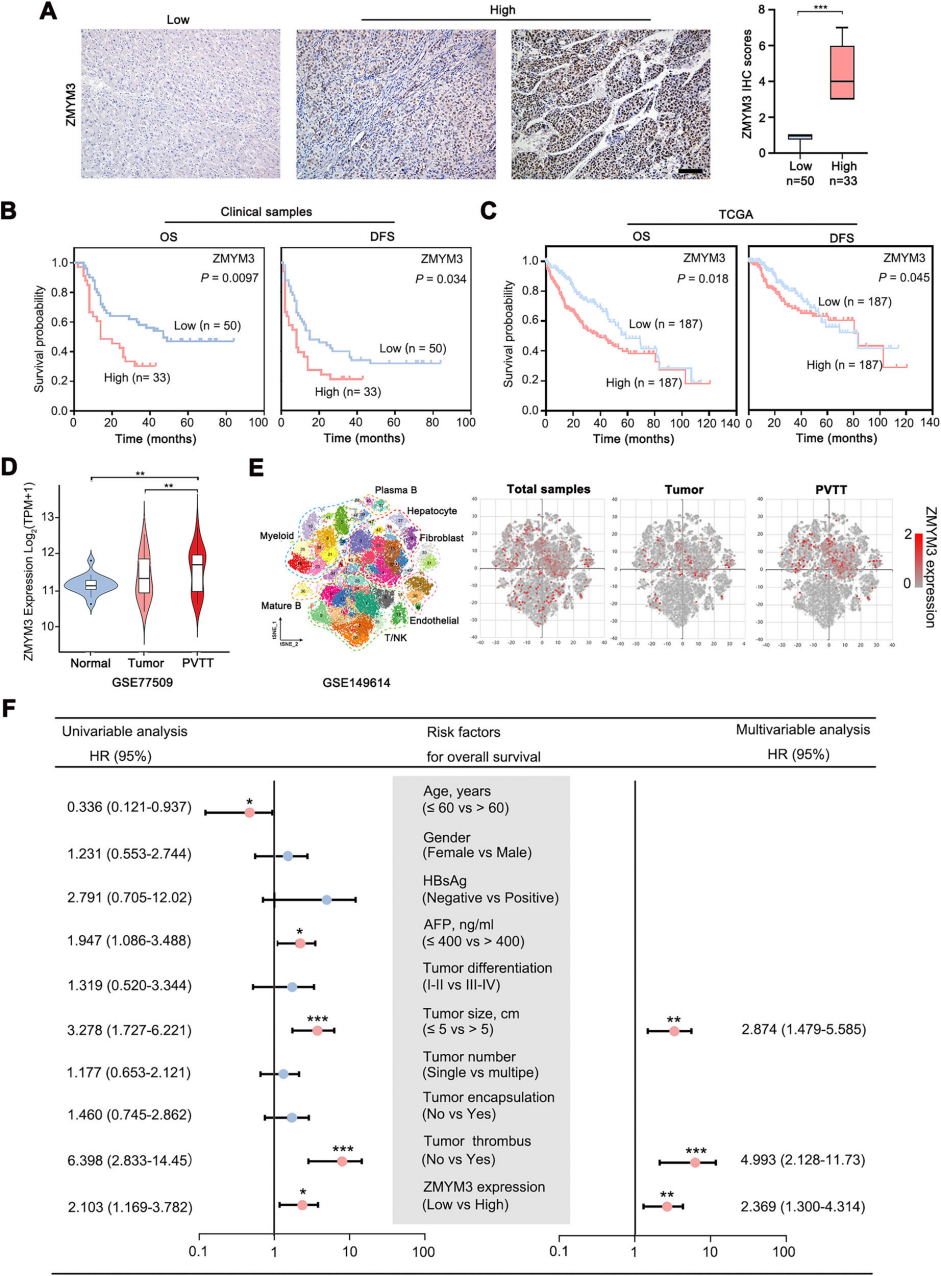
High ZMYM3 expression implied poor prognosis and a heightened invasive capacity in HCC. **A** Representative IHC staining images of ZMYM3 in 83 HCC tissues. Scale bar, 100 μm. The proportion of high and low ZMYM3 expression in HCC tissues was shown in the right panel. **B** Overall survival (OS) and disease-free survival (DFS) curves of 83 HCC patients with high or low ZMYM3 expression. **C** OS and DFS curves of 374 HCC patients from TCGA with high or low ZMYM3 expression. **D** ZMYM3 mRNA expression in adjacent non-tumor tissues, HCC tissues, and portal vein tumor thrombus (PVTT) tissues from the same patient (GSE77509, *n* = 19). **E** Analyzed single-cell RNA sequencing data from primary HCC and PVTT tissues to compare ZMYM3 mRNA expression levels between primary HCC and PVTT tissues (GSE149614). **F** Univariate and multivariate Cox regression analysis of clinicopathological factors associated with overall survival in 83 HCC patients. *P < 0.05; **P < 0.01; ***P < 0.001.

To further investigate the clinical significance of ZMYM3 in hepatocellular carcinoma (HCC), we classified 83 HCC samples into two groups based on IHC scores of ZMYM3 **(high ZMYM3 expression VS low ZMYM3 expression;** Fig. 2A**)**. Kaplan-Meier survival analysis demonstrated that patients with high ZMYM3 expression exhibited significantly reduced overall survival (OS) and disease-free survival (DFS) compared to those with low expression **(**Fig. 2B**)**. These findings were consistent with TCGA data analysis, which also indicated decreased OS and DFS associated with high ZMYM3 expression **(**Fig. 2C**)**.

It is well established that tumor thrombosis is closely linked to the invasion and metastasis of HCC. Specifically, HCC frequently leads to the formation of portal vein tumor thrombosis (PVTT), which facilitates vascular invasion and promotes disease progression and metastasis [22]. Consequently, we analyzed transcriptome sequencing data from normal liver, in situ HCC, and PVTT tissues (GSE77509). Notably, ZMYM3 expression was significantly elevated in PVTT tissues compared to adjacent non-tumorous and HCC tissues **(**Fig. 2D**)**. Furthermore, single-cell RNA sequencing (ScRNA-seq) data (GSE149614) revealed that ZMYM3 expression was also heightened in HCC cells derived from PVTT compared to those from in situ tumor tissues **(**Fig. 2E**)**. This finding demonstrates a significant association between ZMYM3 expression and the invasive/metastatic properties of HCC. Furthermore, univariate and multivariate Cox regression analyses robustly identified ZMYM3 expression as an independent prognostic indicator for both OS and DFS in HCC patients **(**Fig. 2F**;** Supplementary Fig. 1 and Supplementary Tables 3 & 4**)**. These results suggest that ZMYM3 is significantly overexpressed in HCC tissues, with elevated expression levels serving as robust indicators of enhanced tumor invasiveness and unfavorable patient prognosis.

### ZMYM3 promotes the proliferation and inhibits the apoptosis of HCC cells

**Fig. 3:**
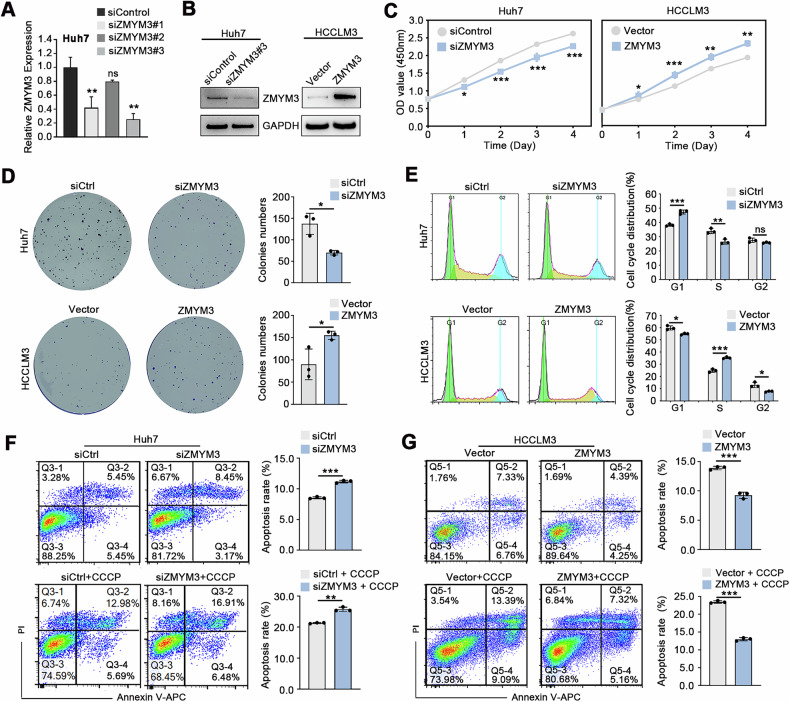
ZMYM3 facilitated the proliferation and suppressed the apoptosis of HCC cells. **A** The knockdown efficiency of ZMYM3-siRNA was verified via qPCR in Huh7 cells. **B** Western blotting was used to validate the knockdown of ZMYM3 in Huh7 cells and the overexpression of ZMYM3 in HCCLM3 cells. **C, D** ZMYM3 knockdown inhibited proliferation and colony formation of HCC cells, while ZMYM3 overexpression promoted proliferation and colony formation of HCC cells. **E** Cell cycle analysis revealed the impact of ZMYM3 knockdown or overexpression on cell cycle progression. **F**, **G** Apoptosis assays revealed that ZMYM3 knockdown enhanced apoptosis of HCC cells, while ZMYM3 overexpression reduced it, under conditions with or without CCCP. *P < 0.05; **P < 0.01; ***P < 0.001.

To further investigate the biological role of ZMYM3 in HCC, we downregulated ZMYM3 with siRNA in Huh7 cells, which naturally exhibit high ZMYM3 expression, and overexpressed ZMYM3 with lentivirus overexpression vector in HCCLM3 cell, which have low endogenous ZMYM3 levels **(**Fig. 3A, B**)**. Cell proliferation and colony formation assays demonstrated that reducing ZMYM3 expression inhibited the growth and colony-forming capacity of HCC cells, whereas overexpression of ZMYM3 promoted proliferation and colony formation in HCC cells **(**Fig. 3C, D**)**.

Subsequently, we employed flow cytometry to assess the effects of ZMYM3 on cell cycle progression and apoptosis. Cell cycle analysis revealed that ZMYM3 downregulation in HCC cells led to an accumulation of cells in the G1 phase and a decrease in the S phase population. Conversely, ZMYM3 upregulation resulted in a reduction in G1 and G2 phase cells and an increase in S phase cells **(**Fig. 3E**)**, indicating that ZMYM3 stimulates HCC cell proliferation. Furthermore, apoptosis assays showed that ZMYM3 downregulation increased the apoptotic rate in HCC cells, while its upregulation decreased the apoptotic rate **(**Fig. 3F**)**. Consistent with these findings, when HCC cells with altered ZMYM3 expression were treated with the apoptosis inducer carbonyl cyanide m-chlorophenylhydrazone (CCCP), ZMYM3 downregulation enhanced CCCP-induced apoptosis, whereas ZMYM3 upregulation attenuated it **(**Fig. 3G**)**. These results suggest that ZMYM3 has an inhibitory effect on apoptosis in HCC cells. Collectively, our findings indicate that ZMYM3 promotes proliferation and inhibits the apoptosis of HCC cells.

### ZMYM3 enhances the invasion and metastasis of HCC cells

**Fig. 4:**
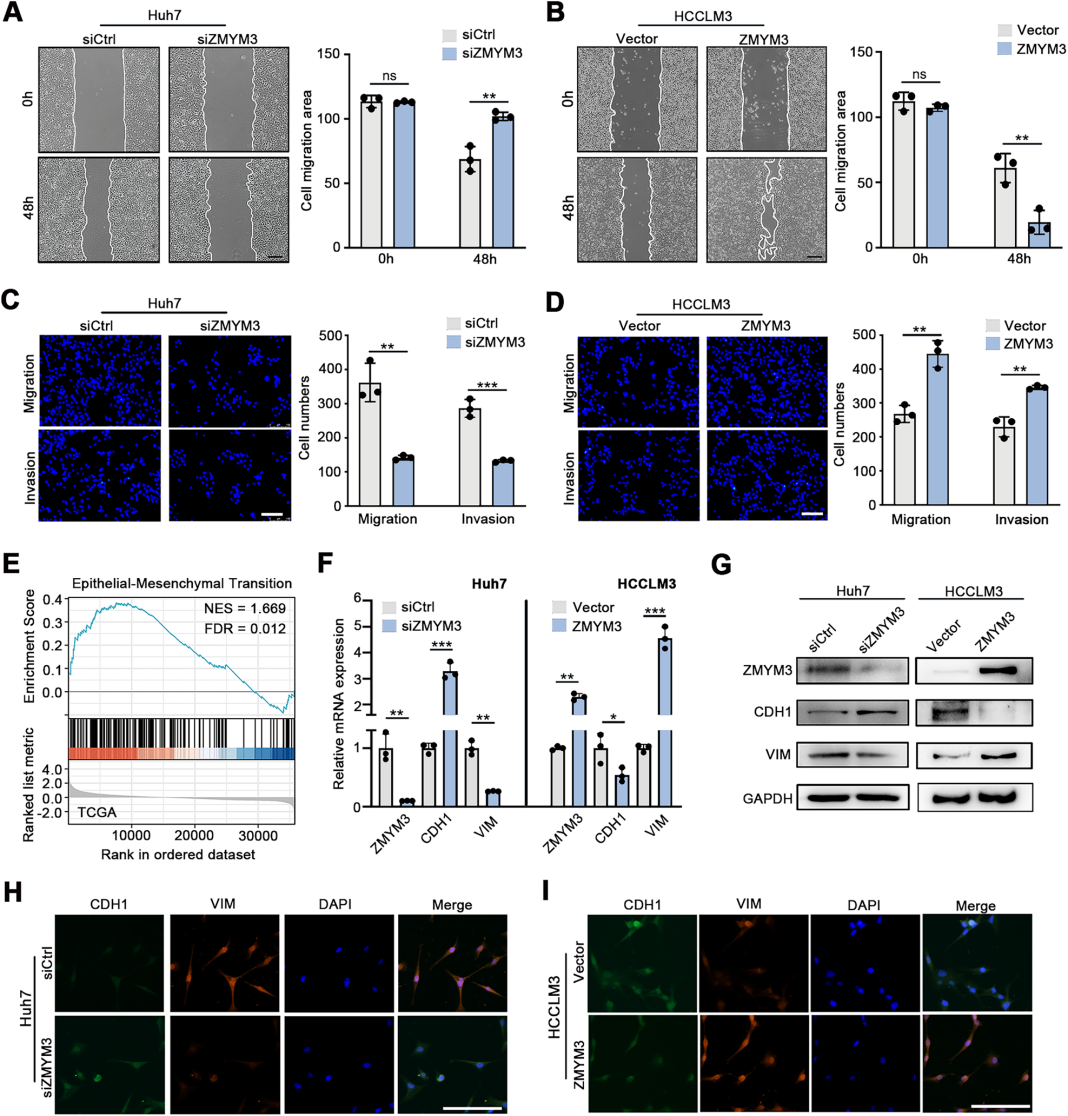
ZMYM3 enhanced the invasion capacity and promoted EMT of HCC cells. **A, B** Wound healing assays were performed to evaluate the effect of ZMYM3 knockdown or overexpression on the migratory capacity of HCC cells. Scale bar, 100 μm. **C, D** Transwell migration and Matrigel invasion assays were performed for 36 h to evaluate the effect of ZMYM3 knockdown or overexpression on the migration and invasion capacity of HCC cells. Scale bar, 100 μm. **E** The GSEA of TCGA data demonstrated that the EMT gene set is significantly enriched in HCC tissues with high ZMYM3 expression. **F–I** Expression of EMT markers ACTA2 and VIM were detected by qPCR **F**, Western blot **G**, and IF staining **H, I** in the indicated cells. Scale bar, 100 μm. *P < 0.05; **P < 0.01; ***P < 0.001.

HCC is highly aggressive and prone to hepatic and extrahepatic metastasis [23]. To explore the role of ZMYM3 in this process, we examined its impact on HCC cell invasion and metastasis. Wound healing assays showed that ZMYM3 knockdown reduced the migration of Huh7 cells, whereas ZMYM3 overexpression enhanced migration in HCCLM3 cells **(**Fig. 4A, B**)**. Transwell migration and Matrigel invasion assays further confirmed that ZMYM3 knockdown inhibited migration and invasion in HCC cells, while ZMYM3 overexpression promoted these processes **(**Fig. 4C, D, Supplement Fig. 2**)**.

Previous studies have demonstrated that epithelial-mesenchymal transition (EMT) plays a crucial role in promoting hepatocellular carcinoma (HCC) cell proliferation, invasion, and metastasis [24]. Our Gene Set Enrichment Analysis (GSEA) of TCGA data revealed significant enrichment of EMT-related gene signatures in HCC tissues with high ZMYM3 expression, implying a regulatory role of ZMYM3 in EMT **(**Fig. 4E**)**. Furthermore, RT-qPCR, Western blot, and immunofluorescence assays confirmed that ZMYM3 regulated the expression of EMT markers. ZMYM3 knockdown increased E-cadherin and decreased Vimentin expression in HCC cells, while ZMYM3 overexpression exhibited the opposite effects **(**Fig. 4F–I**)**. Together, these findings indicate that ZMYM3 promotes EMT and enhances the invasive and metastatic potential of HCC cells.

### ZMYM3 regulates invadopodia formation of HCC cells

**Fig. 5:**
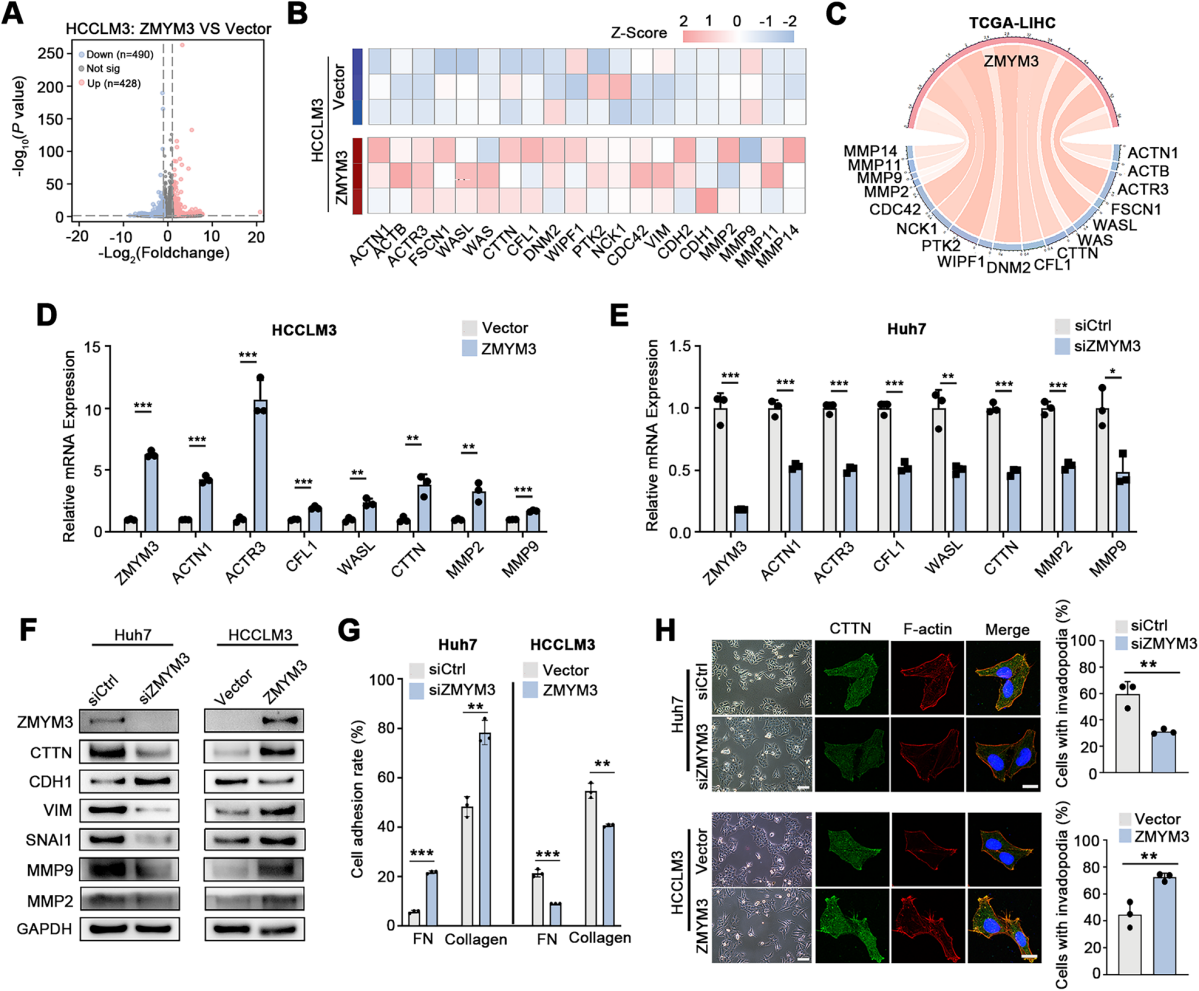
ZMYM3 promoted invadopodia formation of HCC cells. **A** Volcano plot depicting differentially expressed genes (DEGs) in HCC cells with or without ZMYM3 overexpression detected by RNA-seq. **B** The heatmap showed the upregulated invadopodia formation related genes detected by RNA-seq in ZMYM3 overexpressing HCC cells. **C** Circle plots illustrated the correlation between ZMYM3 and invadopodia formation related genes in TCGA data. **D** The expression changes of the invadopodia formation related genes were detected by qPCR in ZMYM3 knockdown HCC cells. **E** The expression changes of the invadopodia formation related genes were detected by qPCR in ZMYM3 over expressing HCC cells. **F** Western blotting detected the expression of the indicated proteins in ZMYM3 knockdown or overexpressing HCC cells. **G** Cell adhesion assays to assess the impact of ZMYM3 knockdown or overexpressing on cell adhesion capacity of HCC cells. **H** Immunofluorescent staining suggested that ZMYM3 knockdown inhibited invadopodia formation, while ZMYM3 overexpression promoted invadopodia formation. Scale bar for bright field images, 100 μm. Scale bar for immunofluorescence images, 20 μm. **P < 0.01; ***P < 0.001.

To investigate the mechanism by which ZMYM3 overexpression promotes malignant biological behaviors of HCC cells, we performed RNA-seq to compare the gene expression profile of control and ZMYM3-overexpressing HCCLM3 cells. Differential expression analysis identified 918 significantly altered genes (|log2FC | >1, *P* < 0.05), with 490 upregulated and 428 downregulated genes **(**Fig. 5A**)**. Functional enrichment revealed involvement in key pathways including MAPK/ERK signaling, cell adhesion, ECM interactions, EMT, proliferation, and migration **(**Supplementary Fig. 3**)**. In addition, our RNA-seq also indicated that ZMYM3 expression was positively correlated with invadopodia formation-related genes **(**Fig. 5B**)**. Invadopodia are actin-rich protrusions of tumor cells which facilitate ECM degradation and metastasis [25]. Consistent with this, correlation analysis of TCGA LIHC data also showed a significant positive association between ZMYM3 and genes involved in invadopodia formation **(**Fig. 5C**)**. Further qPCR confirmed that ZMYM3 knockdown reduced, while overexpression increased, the expression of these genes **(**Fig. 5D, E**)**. Moreover, western blot analysis demonstrated that ZMYM3 expression induced epithelial-mesenchymal transition (EMT) and upregulated matrix metalloproteinases (MMP2 and MMP9), as well as CTTN, a key regulator of invadopodia formation **(**Fig. 5F**)**.

We next evaluated the effects of ZMYM3 on cell adhesion and invadopodia formation. Knockdown of ZMYM3 enhanced cell adhesion, whereas its overexpression reduced adhesion, consistent with a role in promoting ECM degradation and facilitating invasion and migration **(**Fig. 5G**)**. Morphological changes were evident in ZMYM3-modulated cells. ZMYM3 overexpression in HCCLM3 cells resulted in looser cell packing, diminished cell–cell contacts, and elongated or spindle-like morphology, which were reversed upon knockdown in Huh7 cells **(**Fig. 5H**)**. Immunofluorescence staining of cytoskeleton and invadopodia markers showed that ZMYM3 knockdown suppressed, while its overexpression increased, invadopodia formation and cytoskeletal reorganization **(**Fig. 5H**)**. Together, these findings indicate that ZMYM3 enhances HCC cell invasion and migration by driving EMT and invadopodia formation.

### ZMYM3 upregulates CTTN expression by binding to its promoter

**Fig. 6:**
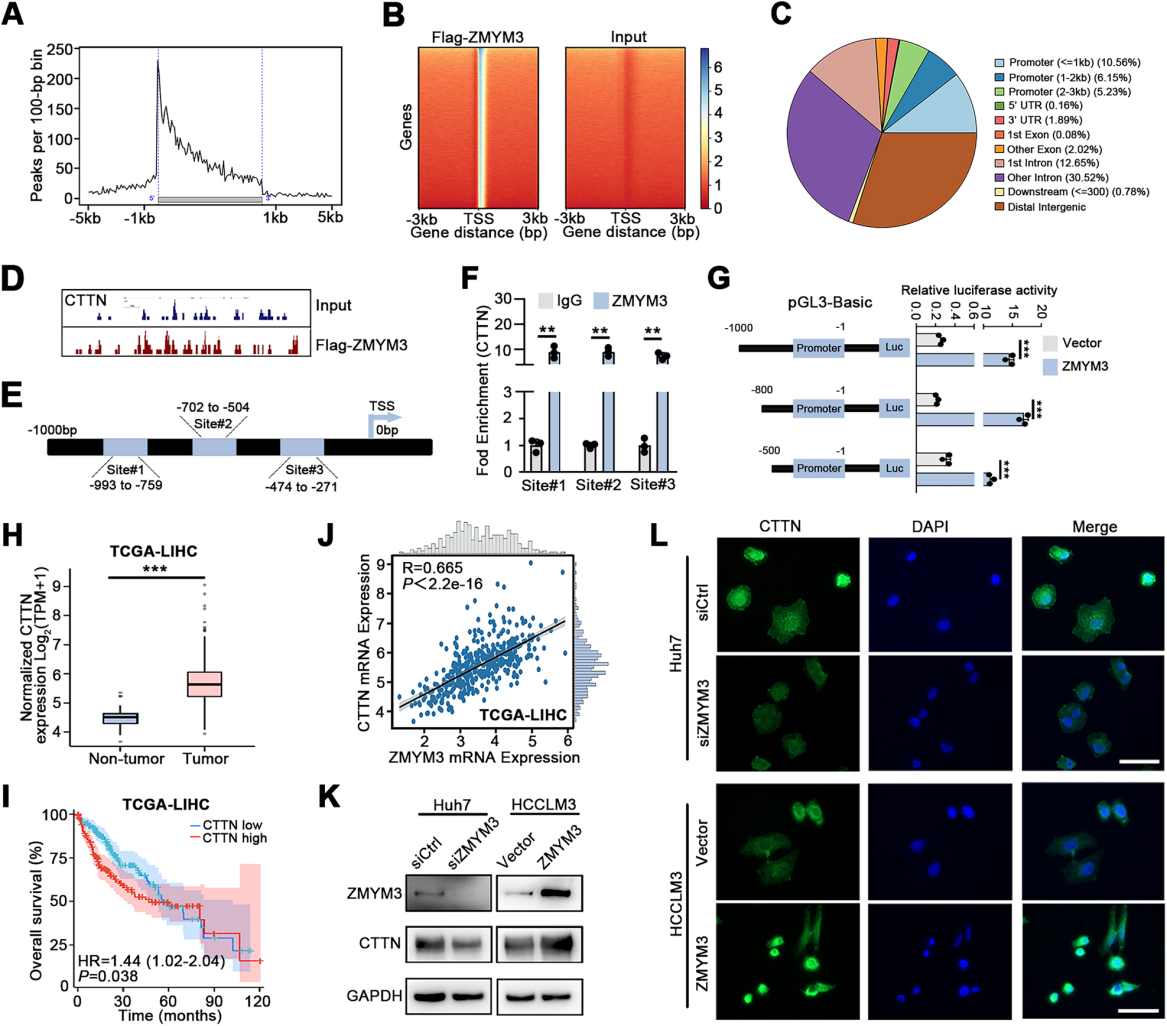
ZMYM3 directly enhanced the transcriptional activity of CTTN. **A** Quantitative analysis of enriched peaks at the proximal end of the TSS. **B** Quantitative analysis of the enrichment intensity of peak-annotated genes at the proximal region of the TSS. **C** Proportional distribution of peaks across gene structural elements. **D** Differences in the distribution and intensity of enriched peaks in CTTN gene in ChIP samples versus control input samples. **E** Binding sites between the transcription factor ZMYM3 and the CTTN promoter based on ChIP-seq data. **F** ChIP-qPCR confirmed the enrichment of the binding sites. **G** Differences in the distribution and intensity of enriched peaks within the genomic region of the CTTN gene between ChIP samples and control input samples. **H** Differential expression of CTTN mRNA between HCC and adjacent non-tumor tissues from TCGA database. **I** OS curves of HCC patients from TCGA with high or low CTTN expression. **J** Correlation analysis between ZMYM3 and CTTN expression in TCGA samples. **K, L** ZMYM3-mediated upregulation of CTTN expression was confirmed through Western blot **I** and IF **J**. Scale bar, 100 μm. **P < 0.01; ***P < 0.001.

To identify the downstream target genes directly regulated by the transcription factor ZMYM3, we performed chromatin immunoprecipitation (ChIP) sequencing assays in HCCLM3 cells overexpressing FLAG-tagged ZMYM3. The ChIP-sequencing revealed a significant enrichment of peaks in regions proximal to the transcription start site (TSS) compared to more distal regions **(**Fig. 6A**)**. When comparing ChIP samples with the input control, gene-annotated peaks displayed higher enrichment intensity near the TSS, consistent with the distribution pattern observed in the heatmap **(**Fig. 6B**)**. Annotation of the genomic structural elements associated with these peaks demonstrated their predominant localization within promoter and intron regions **(**Fig. 6C**)**. In addition, we found that among the key genes involved in the regulation of invadopodia formation, ZMYM3 could directly bind to the promoter of CTTN **(**Fig. 6D**)**. To validate whether ZMYM3 directly regulates CTTN expression through transcriptional binding, we performed ChIP-qPCR and dual-luciferase reporter assays. Leveraging our ChIP-sequencing data, we annotated three putative ZMYM3 binding sites (site#1-3) within the CTTN promoter region **(**Fig. 6E**)**. ChIP-qPCR analysis confirmed significant ZMYM3 enrichment at these sites, thereby establishing CTTN as a direct transcriptional target of ZMYM3 **(**Fig. 6F**)**. Dual-luciferase reporter assays further demonstrated that ZMYM3 binding correlates with transcriptional activity in the promoter region of CTTN. The plasmid vector containing all three ZMYM3 binding sites (sites 1-3) exhibited significantly greater luciferase activity compared to the control plasmid pGL3-basic, indicating strong promoter activity in this region. Notably, the vector containing only binding sites 2 and 3 demonstrated comparable luciferase activity to the full-length construct (sites 1-3). A vector harboring only site 1 also showed significantly elevated activity relative to the control. However, truncation of both sites 1 and 2 resulted in a marked reduction in luciferase activity. Collectively, these findings confirm ZMYM3 as a transcriptional regulator of CTTN and demonstrate that binding sites 2 and 3 within the CTTN promoter are critical for maximal promoter activity **(**Fig. 6G**)**.

TCGA data suggested that CTTN expression was significantly elevated in HCC compared to adjacent non-tumor liver tissues **(**Fig. 6H**)**, and high CTTN expression was associated with poor prognosis of HCC patients **(**Fig. 6I**)**. Correlation studies further indicated a significant positive relationship between ZMYM3 and CTTN expression **(**Fig. 6J**)**. Further experiments demonstrated that knockdown of ZMYM3 downregulated CTTN expression, while ZMYM3 overexpression upregulated CTTN in HCC cells **(**Fig. 6K, L). These results collectively confirmed that ZMYM3 regulated CTTN expression. Thus, ZMYM3 may enhance the invasiveness and metastatic potential of HCC cells by directly upregulating CTTN and promoting invadopodia formation.

### ZMYM3 promotes HCC proliferation and metastasis by upregulating CTTN

**Fig. 7:**
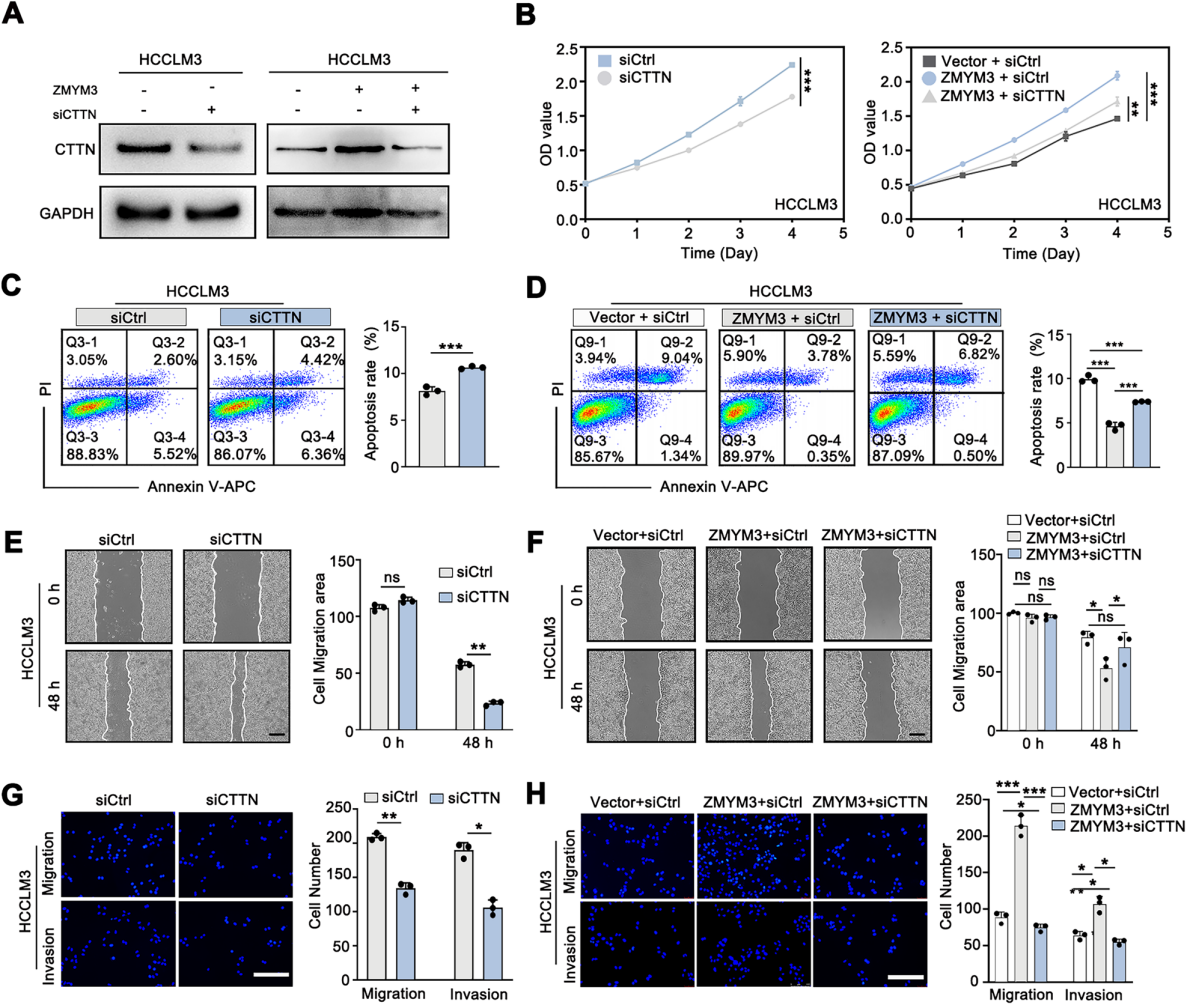
ZMYM3 promoted the proliferation and invasion of HCC cells via upregulating CTTN. **A** CTTN knockdown in HCCLM3 cells with or without ZMYM3 overexpression. **B** CTTN knockdown suppressed HCC cells proliferation and reversed the proliferative-promoting effects induced by ZMYM3 overexpression. **C** Apoptosis assays revealed that CTTN knockdown enhanced apoptosis of HCC cells. **D** CTTN knockdown inhibited the anti-apoptosis effects of ZMYM3 overexpression. **E, F** Wound healing assays indicated that CTTN knockdown suppressed HCC cells migration and reversed the migration-promoting effects induced by ZMYM3 overexpression. Scale bar, 100 μm. **G, H** Transwell assays indicated that CTTN knockdown suppressed migration and invasion capacity of HCC cells and reversed the migration and invasion-promoting effects of ZMYM3 overexpression. Scale bar, 100 μm. *P < 0.05; **P < 0.01; ***P < 0.001; ns not significant.

To determine whether ZMYM3 exerts its effects on HCC cell proliferation, invasion, and metastasis through regulating CTTN expression, we knocked down CTTN in ZMYM3-overexpressing HCC cells **(**Fig. 7A**)**. Cell proliferation assays demonstrated that CTTN knockdown decreased proliferation of HCCLM3 cells and reversed the enhanced proliferation induced by ZMYM3 overexpression in HCCLM3 cells **(**Fig. 7B**)**. Apoptosis assays showed an increase in the apoptotic rate following CTTN knockdown, suggesting that CTTN suppressed apoptosis in HCCLM3 cells and reversed the apoptosis suppression mediated by ZMYM3 overexpression **(**Fig. 7C, D). In addition, wound healing and Transwell assays further revealed that CTTN knockdown not only abolished the promotive effects of ZMYM3 overexpression on cell migration and invasion but also significantly attenuated baseline migratory and invasive capacities **(**Fig. 7E–H**)**. Together, these results indicate that ZMYM3 upregulates CTTN expression, thereby promoting proliferation, migration and invasion in HCC cells.

### ZMYM3 promotes the growth and metastasis of HCC in vivo

**Fig. 8:**
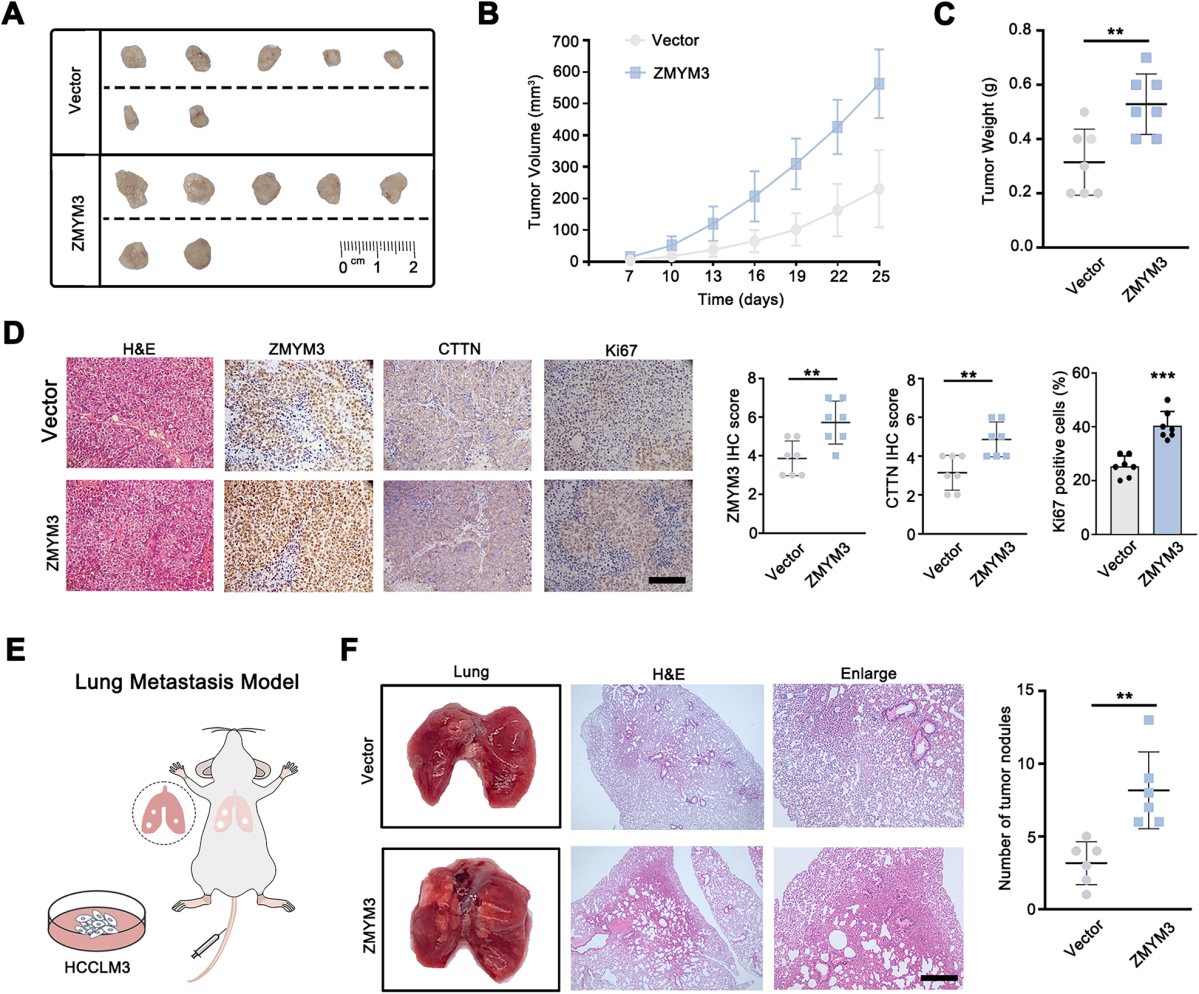
ZMYM3 promoted the growth and metastasis of HCC in vivo. **A** ZMYM3 upregulation enhanced the growth of subcutaneously transplanted HCC in nude mice. **B** Tumor growth curves depicted the changes in tumor volume across each experimental group. **C** Tumor weight differences were observed between the two groups. **D** Representative images showed H&E and IHC staining of Ki67, ZMYM3, and CTTN in xenograft tumors, along with quantitative analysis. Scale bar, 100 μm. **E, F** ZMYM3 upregulation promoted the formation of pulmonary metastatic nodules in nude mice. Scale bar, 100 μm. **P < 0.01; ***P < 0.001.

Based on our in vitro experiments, we have determined that ZMYM3 enhanced the proliferation, invasion, and metastasis of HCC cells. To investigate the in vivo functions of ZMYM3, we conducted subcutaneous xenograft and tail vein-injected lung metastasis mouse models. The tumor growth curve and weight showed that ZMYN3 overexpression significantly promoted the growth of HCC xenografts **(**Fig. 8A–C**)**. Immunohistochemical staining in the samples of xenograft tumors detected an upregulation of CTTN in ZMYM3 overexpression cells **(**Fig. 8D**)**. Moreover, we assessed Ki67 expression level by immunohistochemistry in the xenografts. It showed that Ki67 was upregulated in the xenografts from ZMYM3 overexpressed cells compared with the control cells **(**Fig. 8D**)**. Subsequently, the ZMYM3-overexpressing HCC cells were employed to establish a lung metastasis model via tail vein injection (Fig. 5E). Notably, mice inoculated with these ZMYM3-overexpressing cells exhibited a significantly higher incidence of metastatic nodules in the lungs **(**Fig. 8F**)**. These results indicated that ZMYM3 promoted HCC growth and metastasis.

## Discussion

ZMYM3 is a gene that encodes a zinc finger structure domain protein, which acts as a transcription factor to regulate the expression of related genes. It has been reported to be highly expressed in aggressive cancers including prostate cancer, lung cancer, and breast cancer, and is closely associated with poor prognosis of these types of cancer [13–15]. However, the molecular function of ZMYM3 in HCC is still unclear.

Through analysis of public databases and our own data, we found that ZMYM3 was aberrantly upregulated in HCC tissues, and high expression of ZMYM3 was significantly associated with poor prognosis in HCC patients. Further analysis indicated that ZMYM3 expression in tumor cells within portal vein tumor thrombi (PVTT) was significantly higher than that in primary tumor cells. Since PVTT was closely related to metastasis risk, this result suggested that ZMYM3 may promote the invasion and metastasis of HCC. Out in vitro and in vivo experiments confirmed this conclusion, showing that ZMYM3 promoted the proliferation and invasion of HCC cells.

Research on ZMYM3 in cancer is still limited. Although some studies have reported its potential oncogenic role in various tumors [14, 26], the downstream genes and mechanisms by which ZMYM3 regulates malignant biological behaviors of tumor cells remain unclear. Our study, through transcriptome sequencing and ChIP sequencing of ZMYM3-overexpressing HCC cells, revealed that ZMYM3 regulated key signaling pathways such as WNT, JAK/STAT, and PI3K/AKT. The downstream genes of ZMYM3 were involved in various biological processes, including EMT, cell adhesion, cell growth, cell migration, and cytoskeletal protein synthesis and assembly. These pathways contribute to enhanced proliferation and invasion of HCC cells [27–29]. It suggested that ZMYM3 may promote HCC invasion and metastasis by regulating downstream signaling pathways. Furthermore, we found that ZMYM3 directly upregulated CTTN gene transcription by binding on the CTTN promoter region. The CTTN gene encodes cortactin, a key protein that mediates invadopodia formation in tumor cells. CTTN promotes actin polymerization and assembly by binding to the Arp2/3 complex and F-actin, enhancing tumor cell invasion through cytoskeletal stability and dynamic remodeling [30]. CTTN and its regulation of invadopodia formation play critical roles in HCC invasion and metastasis. Overexpression of CTTN was closely related to tumor thrombi, metastatic HCC, and patient prognosis [31], and studies have shown that in vivo injection of CTTN-overexpressing HCC cells into the liver results in increased intrahepatic metastatic nodules [32]. Our study suggested that CTTN was an important downstream gene through which ZMYM3 exerted its oncogenic effects. ZMYM3 enhanced HCC cell invadopodia formation and promotes invasion and metastasis by directly upregulating CTTN transcription.

Interestingly, most previous studies on ZMYM3 have focused on its role as a component of the LSD1-containing transcriptional repressor complex [33]. Through this mechanism, ZMYM3 has been shown to mediate gene repression, whereas our results reveal that ZMYM3 instead upregulates CTTN expression, in contrast to earlier findings. Previous studies have also characterized ZMYM3 primarily as a scaffold protein within the LSD1 repressor complex [33]. Beyond this, other studies have demonstrated that ZMYM3, as a scaffold protein, can mediate the recruitment of diverse proteins to specific genomic loci to perform distinct functions—for instance, recruiting BRCA1 to DNA double-strand break sites to facilitate repair [15], or mediating the localization of RNase H2 to clear RNA embedded in the genome [17]. Based on these observations, we propose that ZMYM3 may likewise function as a scaffold to recruit proteins with gene-activating roles to defined chromatin regions, thereby promoting the transcription of specific genes such as CTTN under particular cellular conditions. Although this hypothesis requires further validation, our findings provide valuable insights and a promising direction for future investigation.

Additionally, invadopodia formation is closely related to EMT [34, 35]. Invadopodia are actin-rich protrusions of the plasma membrane. Invadopodia promote extracellular matrix (ECM) degradation by facilitating the secretion of matrix metalloproteinases (MMPs), thus enhancing the migratory and invasive capabilities of tumor cells [36]. Mesenchymal cells utilize invadopodia for their migration and invasion, whereas lamellipodia and filopodia are mostly observed in epithelial cells [10]. Our results also show that ZMYM3 can promote EMT and upregulate the expression of various MMPs. Knockdown of ZMYM3 inhibited EMT, invadopodia formation and MMPs expression, and enhanced HCC cells adhesion. In addition, invadopodia can affect the expression and activity of cell surface adhesion molecules, such as integrins, thereby influencing cell interactions with the ECM and cell migration ability [37, 38]. Moreover, by degrading the ECM, invadopodia facilitate the release of cytokines such as IGF and VEGF, which activate downstream signaling pathways to promote tumor development [39, 40]. It is a complex regulatory network. Our research suggests that ZMYM3 may play a key role in this regulatory network, though further studies are needed to confirm its function.

This study identified the abnormal expression of ZMYM3 in HCC tissues and reported that high expression of ZMYM3 was a marker for poor prognosis of HCC patients. Additionally, our findings demonstrated that ZMYM3, as a transcription factor, directly upregulated CTTN expression to promote invadopodia formation and enhance invasion and metastasis of HCC cells. These discoveries provided valuable insights into the molecular mechanisms underlying the regulation of malignant biological behaviors in HCC by ZMYM3 and contributed to understanding the driving mechanisms of invadopodia formation and metastasis in HCC cells.

## Supplementary information

Supplement material

Supplement Figure 1. Univariate and multivariate Cox regression analysis.

Supplement Figure 2. ZMYM3 overexpression enhanced the migration and invasion capacity of HCC cells.

Supplement Figure 3. Enrichment analysis of the different expression genes between ZMYM3 overexpressing and control HCC cells.

Uncropped WB image

Reproducibility checklist

## Data Availability

The data supporting the findings of this study are available from the corresponding author upon reasonable request. The RNA sequencing data have been deposited in the China National Center for Bioinformation (CNCB), BioProject Number PRJCA050436.

## References

[1] Sung H, Ferlay J, Siegel RL, Laversanne M, Soerjomataram I, Jemal A, et al. Global cancer statistics 2020: GLOBOCAN estimates of incidence and mortality worldwide for 36 cancers in 185 countries. CA: A Cancer J Clin. 2021;71:209–49. 10.3322/caac.21660 33538338

[2] Yang G, Yan H, Tang Y, Yuan F, Cao M, Ren Y, et al. Advancements in understanding mechanisms of hepatocellular carcinoma radiosensitivity: A comprehensive review. Chin J Cancer Res. 2023;35:266–82. 37440829 10.21147/j.issn.1000-9604.2023.03.06 PMC10334493

[3] Oura K, Morishita A, Masaki T. Molecular and Functional Roles of MicroRNAs in the Progression of Hepatocellular Carcinoma-A Review. Int J Mol Sci. 2020;21:8362. 33171811 10.3390/ijms21218362 PMC7664704

[4] Sun B, Ji WD, Wang WC, Chen L, Ma JY, Tang EJ, et al. Circulating tumor cells participate in the formation of microvascular invasion and impact on clinical outcomes in hepatocellular carcinoma. Front Genet. 2023;14:1265866. 38028589 10.3389/fgene.2023.1265866 PMC10652898

[5] Yu Y, Peng XD, Qian XJ, Zhang KM, Huang X, Chen YH, et al. Fis1 phosphorylation by Met promotes mitochondrial fission and hepatocellular carcinoma metastasis. Signal Transd Target Ther. 2021;6:401. 10.1038/s41392-021-00790-2 PMC8632923 34848680

[6] Huang Q, Zhang R, Xia Y, Shen J, Dong H, Li X, et al. DAB2IP suppresses invadopodia formation through destabilizing ALK by interacting with USP10 in breast cancer. iScience. 2023;26:107606. 37664607 10.1016/j.isci.2023.107606 PMC10470318

[7] McNiven MA, Baldassarre M, Buccione R. The role of dynamin in the assembly and function of podosomes and invadopodia. Front Biosci. 2004;9:1944–53. 14977600 10.2741/1348

[8] Thuault S, Ghossoub R, David G, Zimmermann P. A Journey on Extracellular Vesicles for Matrix Metalloproteinases: A Mechanistic Perspective. Front Cell Dev Biol. 2022;10:886381. 35669514 10.3389/fcell.2022.886381 PMC9163832

[9] Brasher MI, Chafe SC, McDonald PC, Nemirovsky O, Gorshtein G, Gerbec ZJ, et al. Syntaxin4-Munc18c Interaction Promotes Breast Tumor Invasion and Metastasis by Regulating MT1-MMP Trafficking. Mol Cancer Res. 2022;20:434–45. 34876482 10.1158/1541-7786.MCR-20-0527 PMC9306282

[10] Karamanou K, Franchi M, Vynios D, Brézillon S. Epithelial-to-mesenchymal transition and invadopodia markers in breast cancer: Lumican a key regulator. Semin Cancer Biol. 2020;62:125–33. 31401293 10.1016/j.semcancer.2019.08.003

[11] Guzzo CM, Ringel A, Cox E, Uzoma I, Zhu H, Blackshaw S, et al. Characterization of the SUMO-binding activity of the myeloproliferative and mental retardation (MYM)-type zinc fingers in ZNF261 and ZNF198. PLoS ONE. 2014;9:e105271. 25133527 10.1371/journal.pone.0105271 PMC4136804

[12] Kojima KK, Jurka J. Crypton transposons: identification of new diverse families and ancient domestication events. Mob DNA. 2011;2:12. 22011512 10.1186/1759-8753-2-12 PMC3212892

[13] Liu W, Zheng SL, Na R, Wei L, Sun J, Gallagher J, et al. Distinct Genomic Alterations in Prostate Tumors Derived from African American Men. Mol Cancer Res. 2020;18:1815–24. 33115829 10.1158/1541-7786.MCR-20-0648

[14] Kudo N, Kudoh S, Matsuo A, Motooka Y, Ito T. ZMYM3 May Promote Cell Proliferation in Small Cell Lung Carcinoma. Acta Histochem Cytochem. 2021;54:143–53. 34764523 10.1267/ahc.21-00012 PMC8569135

[15] Leung JW, Makharashvili N, Agarwal P, Chiu LY, Pourpre R, Cammarata MB, et al. ZMYM3 regulates BRCA1 localization at damaged chromatin to promote DNA repair. Genes Dev. 2017;31:260–74. 28242625 10.1101/gad.292516.116 PMC5358723

[16] Shen H, Chen Z, Ding X, Qi X, Cen J, Wang Y, et al. BMI1 reprogrammes histone acetylation and enhances c-fos pathway via directly binding to Zmym3 in malignant myeloid progression. J Cell Mol Med. 2014;18:1004–17. 24571310 10.1111/jcmm.12246 PMC4508141

[17] Shapson-Coe A, Valeiras B, Wall C, Rada C. Aicardi-Goutières Syndrome associated mutations of RNase H2B impair its interaction with ZMYM3 and the CoREST histone-modifying complex. PLoS ONE. 2019;14:e0213553. 30889214 10.1371/journal.pone.0213553 PMC6424451

[18] Ramos-García P, González-Moles M, González-Ruiz L, Ayén Á, Ruiz-Ávila I, Navarro-Triviño FJ, et al. An update of knowledge on cortactin as a metastatic driver and potential therapeutic target in oral squamous cell carcinoma. Oral Dis. 2019;25:949–71. 29878474 10.1111/odi.12913

[19] Moon SJ, Choi HJ, Kye YH, Jeong GY, Kim HY, Myung JK, et al. CTTN Overexpression Confers Cancer Stem Cell-like Properties and Trastuzumab Resistance via DKK-1/WNT Signaling in HER2 Positive Breast Cancer. Cancers. 2023;15:1168. 10.3390/cancers15041168 PMC9954024 36831511

[20] Chuma M, Sakamoto M, Yasuda J, Fujii G, Nakanishi K, Tsuchiya A, et al. Overexpression of cortactin is involved in motility and metastasis of hepatocellular carcinoma. J Hepatol. 2004;41:629–36. 15464244 10.1016/j.jhep.2004.06.018

[21] Li Y, Fu Y, Hu X, Sun L, Tang D, Li N, et al. The HBx-CTTN interaction promotes cell proliferation and migration of hepatocellular carcinoma via CREB1. Cell Death Dis. 2019;10:405. 31138777 10.1038/s41419-019-1650-x PMC6538608

[22] Datta Gupta SS, Shamim SA, Gamanagatti S, Gupta P, Khan MA, Mallia MB, et al. Re-188 lipiodol in hepatocellular carcinoma with portal vein thrombosis: a pilot study using novel chelating agent N-DEDC and its comparison with (A)HDD. Nucl Med Commun. 2024;45:510–8. 38632971 10.1097/MNM.0000000000001840

[23] Kudo M. Treatment of advanced hepatocellular carcinoma with emphasis on hepatic arterial infusion chemotherapy and molecular targeted therapy. Liver Cancer. 2012;1:62–70. 24159574 10.1159/000342402 PMC3747546

[24] He L, Zhou X, Qu C, Hu L, Tang Y, Zhang Q, et al. Musashi2 predicts poor prognosis and invasion in hepatocellular carcinoma by driving epithelial-mesenchymal transition. J Cell Mol Med. 2014;18:49–58. 24305552 10.1111/jcmm.12158 PMC3916117

[25] Niland S, Riscanevo AX, Eble JA. Matrix Metalloproteinases Shape the Tumor Microenvironment in Cancer Progression. Int J Mol Sci. 2021;23:146. 35008569 10.3390/ijms23010146 PMC8745566

[26] Chang S, Yim S, Park H. The cancer driver genes IDH1/2, JARID1C/ KDM5C, and UTX/ KDM6A: crosstalk between histone demethylation and hypoxic reprogramming in cancer metabolism. Exp Mol Med. 2019;51:1–17. 31221981 10.1038/s12276-019-0230-6 PMC6586683

[27] El-Ashmawy NE, Khedr EG, Abo-Saif MA, Hamouda SM. Long noncoding RNAs as regulators of epithelial mesenchymal transition in breast cancer: A recent review. Life Sci. 2024;336:122339. 38097110 10.1016/j.lfs.2023.122339

[28] Wei N, Wu X, Yu Y, Zhou H, Cui K, Zhao X, et al. CD146 Promotes EMT-Mediated Migration and Invasion of NSCLC via PI3K/Akt Signaling Pathway. Front Biosci. 2024;29:140. 10.31083/j.fbl2904140 38682195

[29] Liang Y, Kong D, Zhang Y, Li S, Li Y, Dong L, et al. Curcumin inhibits the viability, migration and invasion of papillary thyroid cancer cells by regulating the miR-301a-3p/STAT3 axis. Exp Ther Med. 2021;22:875. 34194553 10.3892/etm.2021.10307 PMC8237388

[30] Jeannot P, Besson A. Cortactin function in invadopodia. Small GTPases. 2020;11:256–70. 29172953 10.1080/21541248.2017.1405773 PMC7549685

[31] Zhao HF, Liu YS, Wang J, Wu CP, Zhou XM, Cai LR, et al. Nuclear transport of phosphorylated LanCL2 promotes invadopodia formation and tumor progression of glioblastoma by activating STAT3/Cortactin signaling. J Adv Res. 2024;69:139–55. 38492734 10.1016/j.jare.2024.03.007 PMC11954814

[32] Zhao G, Huang ZM, Kong YL, Wen DQ, Li Y, Ren L, et al. Cortactin is a sensitive biomarker relative to the poor prognosis of human hepatocellular carcinoma. World J Surg Oncol. 2013;11:74. 23518204 10.1186/1477-7819-11-74 PMC3620941

[33] Hu X, Shen B, Liao S, Ning Y, Ma L, Chen J, et al. Gene knockout of Zmym3 in mice arrests spermatogenesis at meiotic metaphase with defects in spindle assembly checkpoint. Cell Death Dis. 2017;8:e2910. 28661483 10.1038/cddis.2017.228 PMC5520888

[34] Martínez-López A, García-Casas A, Infante G, González-Fernández M, Salvador N, Lorente M, et al. POTEE promotes breast cancer cell malignancy by inducing invadopodia formation through the activation of SUMOylated Rac1. Mol Oncol. 2024;18:620–40. 38098337 10.1002/1878-0261.13568 PMC10920093

[35] Quilaqueo-Millaqueo N, Brown-Brown DA, Vidal-Vidal JA, Niechi I. NOX proteins and ROS generation: role in invadopodia formation and cancer cell invasion. Biol Res. 2024;57:98. 39696702 10.1186/s40659-024-00577-z PMC11657503

[36] Venghateri JB, Dassa B, Morgenstern D, Shreberk-Shaked M, Oren M, Geiger B. Deciphering the involvement of the Hippo pathway co-regulators, YAP/TAZ in invadopodia formation and matrix degradation. Cell Death Dis. 2023;14:290. 37185904 10.1038/s41419-023-05769-1 PMC10130049

[37] Mishra YG, Manavathi B. Focal adhesion dynamics in cellular function and disease. Cell Signal. 2021;85:110046. 34004332 10.1016/j.cellsig.2021.110046

[38] Hoshino D, Branch KM, Weaver AM. Signaling inputs to invadopodia and podosomes. J Cell Sci. 2013;126:2979–89. 23843616 10.1242/jcs.079475 PMC3711196

[39] Zhang Z, Yu Y, Liu S, Li J, Zhao B, Wang F, et al. Simultaneous Visualization and Depletion of Peroxynitrite by a Simple Aggregation-Induced Emission Nanoprobe for Preventing Breast Cancer Metastasis after Surgery. Anal Chem. 2024;96:4180–9. 38436249 10.1021/acs.analchem.3c05292

[40] Pan S, Hu Y, Gan L, Lai J, Zheng P, Zhang Y, et al. Matrix metalloproteinase-2 inducing COL1A1 synthesis via integrin alpha Ⅴ promotes invasion and metastasis of cholangiocarcinoma cells. Ann Hepatol. 2024;29:101279. 38123132 10.1016/j.aohep.2023.101279

