# Supplementary material for: Transcriptional factor ZMYM3 promotes hepatocellular carcinoma metastasis by upregulating CTTN and inducing invadopodia formation
Source: Cell Death Dis. 2026 Mar 3;17(1):294. doi: 10.1038/s41419-026-08506-6 (PMC13039738; doi:10.1038/s41419-026-08506-6)
Supplement: Supplementary file 1 — Supplement material [file 41419_2026_8506_MOESM1_ESM.docx]

**Supplement Figure 1. Univariate and multivariate Cox regression analysis.**

Univariate and multivariate Cox regression analysis of clinicopathological factors associated with disease-free survival (DFS) in 83 HCC patients.

**Supplement Figure 2. ZMYM3 overexpression enhanced the migration and invasion capacity of HCC cells.**

Transwell migration and Matrigel invasion assays were performed for 16 hours to evaluate the effect of ZMYM3 knockdown on the migration and invasion capacity of HCC cells. Scale bar, 100 μm. **, P<0.01.

**Supplement Figure 3. Enrichment analysis of the different expression genes between ZMYM3 overexpressing and control HCC cells.**

1. The GO enrichment analysis revealed the biological process entries involved by the different expression genes. **(B)** The KEGG enrichment analysis showed enriched pathways for different expression genes. **(C)** The GSEA demonstrated enriched gene set entries for different expression genes. **(D)** The GSVA displayed enriched gene set entries for different expression genes.

**Supplementary Table 1. Antibodies used in the study**

| **Antibodies** | **Supplier** | **Catalog NO.** | **Working Conditions** | **Application** |
| --- | --- | --- | --- | --- |
| Anti-ZMYM3 | ATLAS ANTIBODIES | Cat #A69501 | 1:200 | IHC |
| Anti-ZMYM3 | Abcam | Cat #Ab19165 | 1:1000 | WB |
| Coractin (CTTN) | Proteintech | Cat #11381-1-AP | 1:200 | IHC |
|  |  |  | 1:200 | IF |
|  |  |  | 1:1000 | WB |
| E-cadherin | Cell Signaling Technology | Cat #3195 | 1:200 | IF |
|  |  |  | 1:1000 | WB |
| Recombinant Anti-Vimentin | Abcam | Cat #ab92547 | 1:200 | IF |
|  |  |  | 1:1000 | WB |
| Recombinant Anti-Ki67 | Abcam | Cat #ab16667 | 1:100 | IHC |
| Anti-SNAIL | Wanlei | Cat #WL01863 | 1:1000 | WB |
| Anti-MMP2 | Wanlei | Cat #WL03224 | 1:1000 | WB |
| Anti-MMP9 | Wanlei | Cat #WL03096 | 1:1000 | WB |
| GAPDH | Cell Signaling Technology | Cat #2118 | 1:4000 | WB |

**Supplementary Table 2. The PCR primer sequences and siRNA sequences used in the study**

| **Name** | **Sequence** |
| --- | --- |
| GAPDH human qPCR forward primer | 5’-ATGACCCCTTCATTGACC-3’ |
| GAPDH human qPCR reverse primer | 5’-GAAGATGGTGATGGGATTTC-3’ |
| ZMYM3 human qPCR forward primer | 5’-ACTAACCCTGCCGCAGTACAT-3’ |
| ZMYM3 human qPCR reverse primer | 5’-GCAGGGTCAATGGGTCAATG-3’ |
| E-cadherin human qPCR forward primer | 5’-TCGACACCCGATTCAAAGTGG-3’ |
| E-cadherin human qPCR reverse primer | 5’-TTCCAGAAACGGAGGCCTGAT-3’ |
| Vimentin human qPCR forward primer | 5’-TGGCCGACGCCATCAACACC-3’ |
| Vimentin human qPCR reverse primer | 5’-CACCTCGACGCGGGCTTTGT-3’ |
| ACTN1 human qPCR forward primer | 5’-CTGGGCATCCACAATGAGGT-3’ |
| ACTN1 human qPCR reverse primer | 5’-CTGTCTTCTTCTGGGGGTCG-3’ |
| ACTR3 human qPCR forward primer | 5’-GGAGTCCGCAGTCTTACGAG-3’ |
| ACTR3 human qPCR reverse primer | 5’-TCTGGAGGACCTGGTAGAGG-3’ |
| CFL1 human qPCR forward primer | 5’- AAGTGGCGGTAGATGGTA-3’ |
| CFL1 human qPCR reverse primer | 5’-TTGTAGCGACTGGATTTT-3’ |
| WASL human qPCR forward primer | 5’-CGCCTCTGACTCCACTTCTC-3’ |
| WASL human qPCR reverse primer | 5’-CTGGACGGAGCTCATGGTTT-3’ |
| CTTN human qPCR forward primer | 5’-GACAAATGTGCCCTTGGCTG-3’ |
| CTTN human qPCR reverse primer | 5’-CTGCCTCTCCGACTGAACAC-3’ |
| MMP2 human qPCR forward primer | 5’-GCTGCATCCAGACTTCCTCA-3’ |
| MMP2 human qPCR reverse primer | 5’-AGGTCCTGGCAATCCCTTTG-3’ |
| MMP9 human qPCR forward primer | 5’-GGACAAGCTCTTCGGCTTCT-3’ |
| MMP9 human qPCR reverse primer | 5’-TCGCTGGTACAGGTCGAGTA-3’ |
| CTTN#site1 human qPCR forward primer | 5’-TTCTTCCAAGTAGAGAGTAAAGCA-3’ |
| CTTN#site1 human qPCR reverse primer | 5’-AGTGCACCCATTTTGATTTCAAGT-3’ |
| CTTN#site2 human qPCR forward primer | 5’-GGCTGTTGCCACAAGGAGG-3’ |
| CTTN#site2 human qPCR reverse primer | 5’-GCTCCGAGGGGGATGTGT-3’ |
| CTTN#site3 human qPCR forward primer | 5’-CGCGGAACAAGCTCTCAGT-3’ |
| CTTN#site3 human qPCR reverse primer | 5’-AACATGGGAGACAAGACCCTG-3’ |
| CTTN#site4 human qPCR forward primer | 5’-TATTTTAGGCCACGCTGGGT-3’ |
| CTTN#site4 human qPCR reverse primer | 5’-GGTCAAGCGGGACAGTCTTC-3’ |
| CTTN#site5 human qPCR forward primer | 5’-CTGGGCTTCTCCTATCCACC-3’ |
| CTTN#site5 human qPCR reverse primer | 5’-ATTCCCACGTCACCTGATGC-3’ |
| CTTN#site6 human qPCR forward primer | 5’-TTCCTGTTGCTGCCGTAGTC-3’ |
| CTTN#site6 human qPCR reverse primer | 5’-GGCCACCAGGAGGAACAC-3’ |
| Human ZMYM3 siRNA target sequence1 | 5’-GGGCTTACATCTACACCAA-3’ |
| Human ZMYM3 siRNA target sequence2 | 5’-GAGGCTCCTTCCATGAGTT-3’ |
| Human ZMYM3 siRNA target sequence3 | 5’-GCTCCAGATTCTCCAATAA-3’ |
| Human CTTN siRNA target sequence | 5’-GAAGCATGCCTCCCAGAAA-3’ |

**Supplementary Table 3. Univariate analysis of factors associated with OS and DFS in 83 patients**

| **Features** | **OS** |  |  | **DFS** |  |
| --- | --- | --- | --- | --- | --- |
|  | **HR (95% CI)** | ***P* value** |  | **HR (95% CI)** | ***P* value** |
| Age (≤60 *vs.* >60)的 | 0.336 (0.121-0.937) | **0.037** |  | 2.162 (0.929-5.035) | 0.074 |
| Gender (Female *vs.* Male) | 1.231 (0.553-2.744) | 0.611 |  | 1.348 (0.638-2.847) | 0.434 |
| HBsAg (Negative *vs.* Positive) | 2.191 (0.705-12.02) | 0.140 |  | 2.284 (0.712-7.319) | 0.165 |
| AFP (≤400 *vs.* >400, ng/ml) | 1.947 (1.086-3.488) | **0.025** |  | 1.844 (1.090-3.104) | **0.021** |
| Tumor differentiation (I-II *vs.* III-IV) | 1.319 (0.520-3.344) | 0.566 |  | 0.889 (0.355-2.226) | 0.802 |
| Tumor size (≤5 cm *vs.* >5 cm) | 3.278 (1.727-6.221) | **< 0.001** |  | 2.688 (1.547-4.673) | **< 0.001** |
| Tumor number (Single *vs.* Multiple) | 1.177 (0.653-2.121) | 0.588 |  | 1.122 (0.820-1.536) | 0.472 |
| Tumor encapsulation (No *vs.* Yes) | 1.460 (0.745-2.862) | 0.270 |  | 1.160 (0.801-1.679) | 0.432 |
| Tumor thrombus (No *vs.* Yes) | 6.398 (2.833-14.45) | **< 0.001** |  | 4.731 (2.135-10.48) | **< 0.001** |
| ZMYM3 expression (Low *vs.* High) | 2.103 (1.169-3.782) | **0.013** |  | 1.706 (1.014-2.871) | **0.044** |

**Supplementary Table 4. Multivariate analysis of factors associated with OS and DFS in 83 patients**

| **Features** | **OS** |  |  | **DFS** |  |
| --- | --- | --- | --- | --- | --- |
|  | **HR (95% CI)** | ***P* value** |  | **HR (95% CI)** | ***P* value** |
| Tumor size (≤5 cm *vs.* >5 cm) | 2.874 (1.479-5.585) | **0.002** |  | 2.440 (1.377-4.327) | **0.002** |
| Tumor thrombus (No *vs.* Yes) | 4.993 (2.128-11.73) | **< 0.001** |  | 3.509 (1.544-7.975) | **0.003** |
| ZMYM3 expression (Low *vs.* High) | 2.369 (1.300-4.314) | **0.004** |  | 1.810 (1.069-3.066) | **0.027** |
