# Supplementary figures and images for: Transcriptional factor ZMYM3 promotes hepatocellular carcinoma metastasis by upregulating CTTN and inducing invadopodia formation
Source: Cell Death Dis. 2026 Mar 3;17(1):294. doi: 10.1038/s41419-026-08506-6 (PMC13039738; doi:10.1038/s41419-026-08506-6)

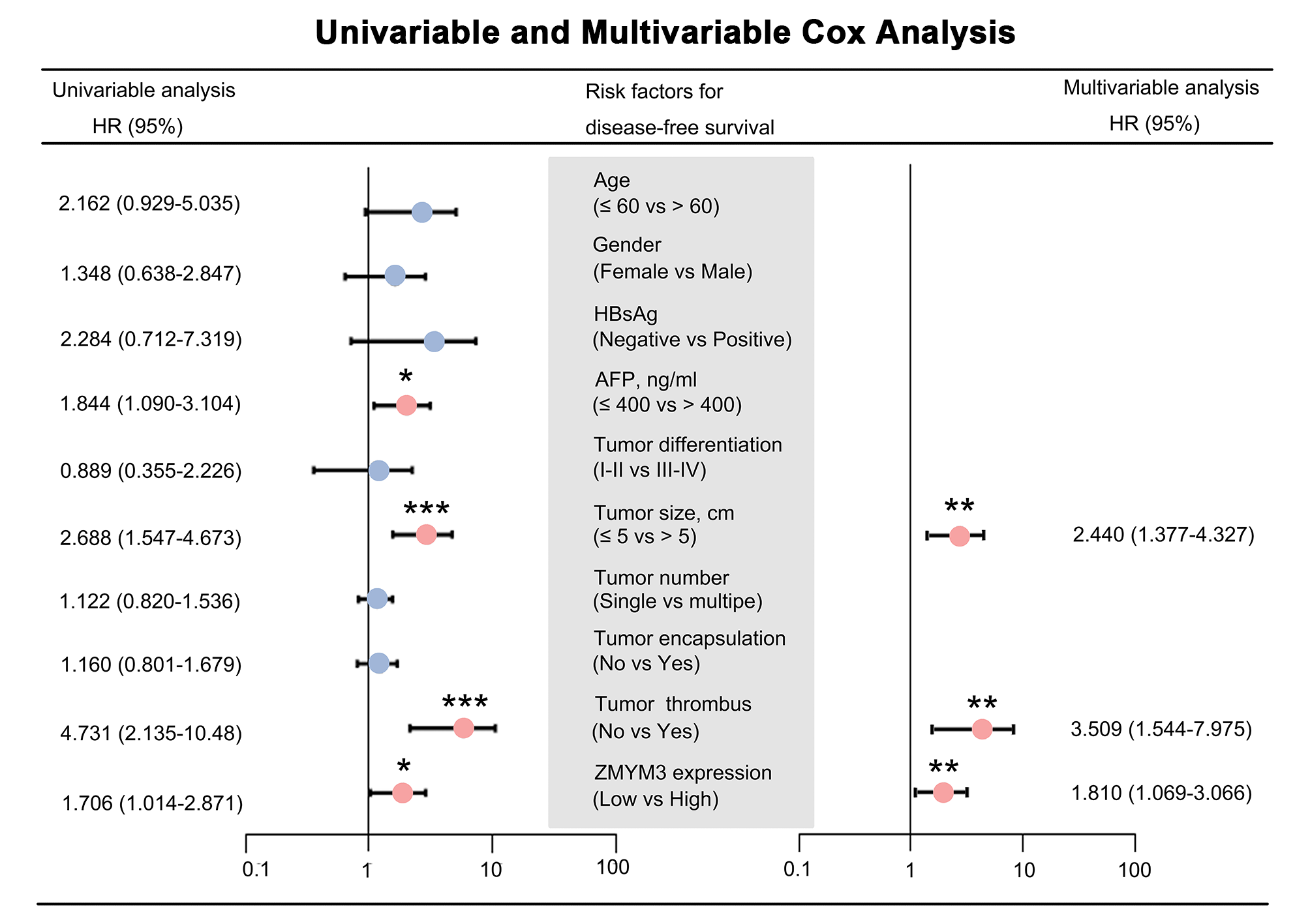

Supplement: Supplementary file 2 — Supplement Figure 1. Univariate and multivariate Cox regression analysis. [file 41419_2026_8506_MOESM2_ESM.tif]

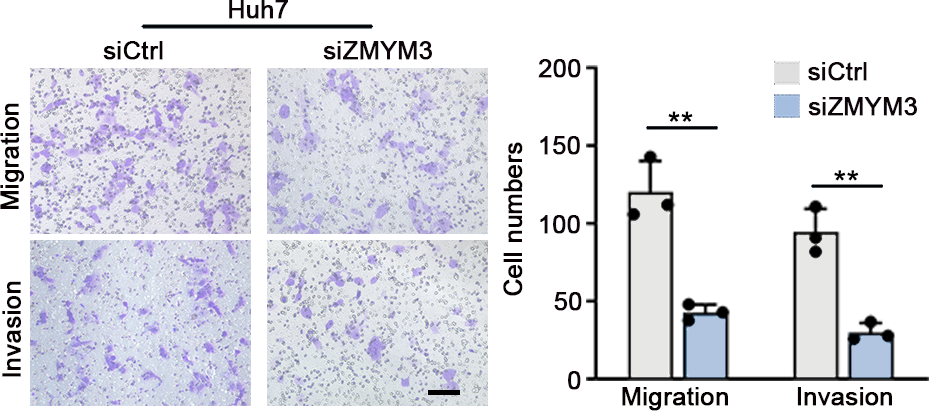

Supplement: Supplementary file 3 — Supplement Figure 2. ZMYM3 overexpression enhanced the migration and invasion capacity of HCC cells. [file 41419_2026_8506_MOESM3_ESM.tif]

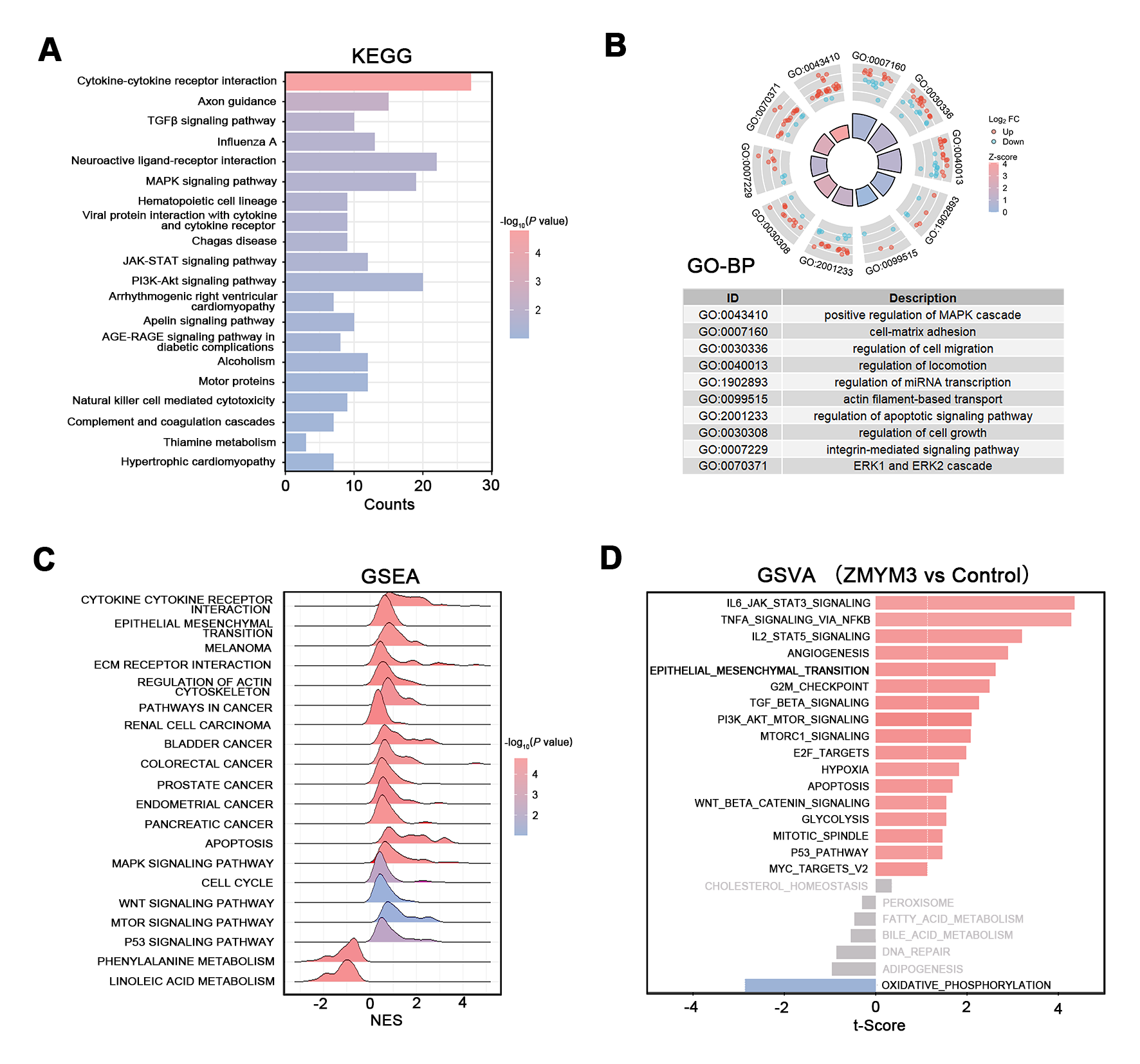

Supplement: Supplementary file 4 — Supplement Figure 3. Enrichment analysis of the different expression genes between ZMYM3 overexpressing and control HCC cells. [file 41419_2026_8506_MOESM4_ESM.tif]

Figure 1G

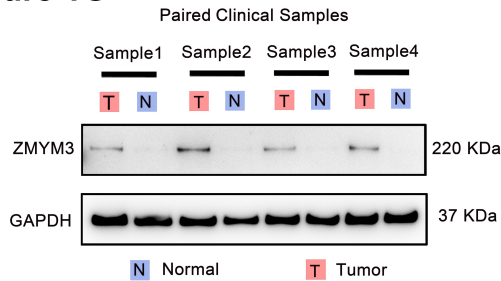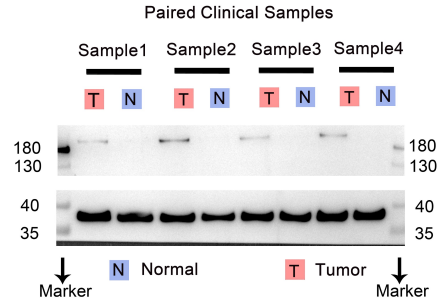

Figure 3B

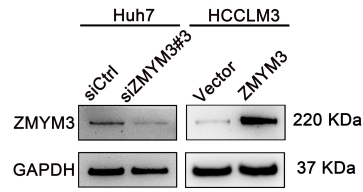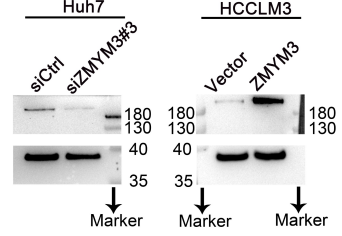

Figure 4G

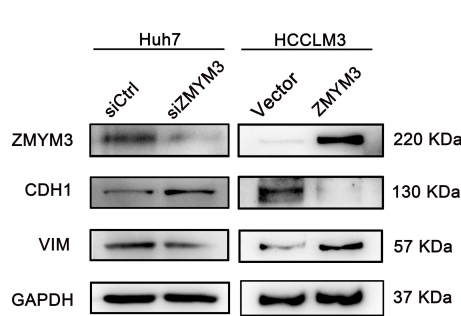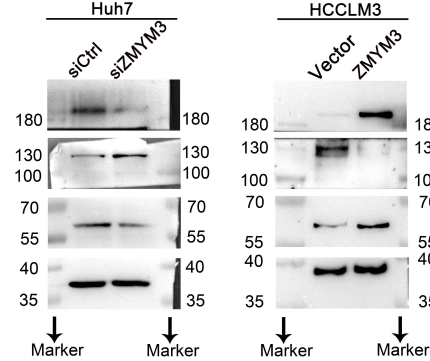

Figure 5F

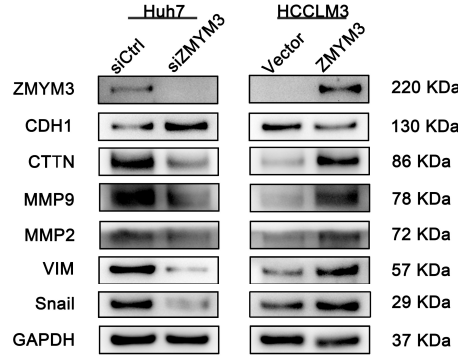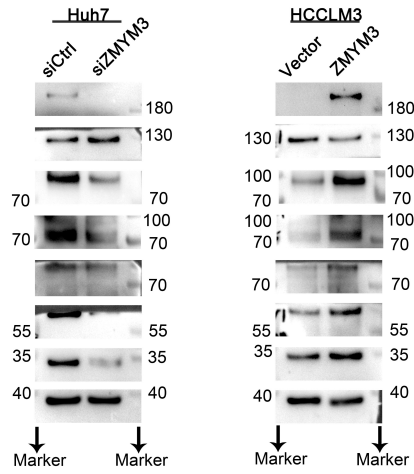

Figure 6K

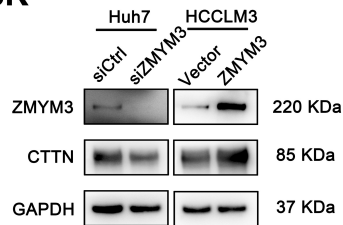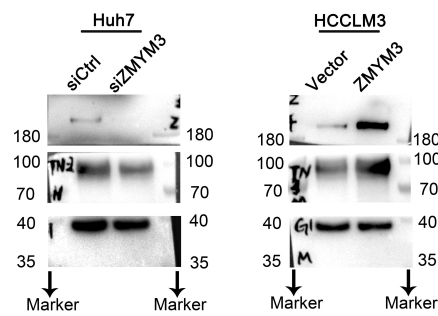

Figure 7A

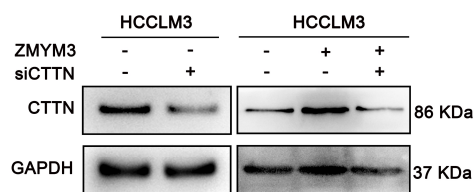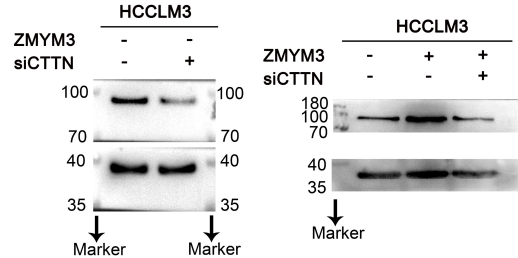

Supplement: Supplementary file 5 — Uncropped WB image [file 41419_2026_8506_MOESM5_ESM.pdf]
